# Immunogenicity, Safety and Efficacy of the Dengue Vaccine TAK-003: A Meta-Analysis

**DOI:** 10.3390/vaccines12070770

**Published:** 2024-07-13

**Authors:** Maria Elena Flacco, Alessandro Bianconi, Giovanni Cioni, Matteo Fiore, Giovanna Letizia Calò, Gianmarco Imperiali, Vittorio Orazi, Marco Tiseo, Anastasia Troia, Annalisa Rosso, Lamberto Manzoli

**Affiliations:** 1 Department of Environmental and Prevention Sciences, School of Public Health, University of Ferrara, Via Fossato di Mortara 44, 44121 Ferrara, Italy; mariaelena.flacco@unife.it (M.E.F.); giovanni01.cioni@edu.unife.it (G.C.); giovannaletizia.calo@edu.unife.it (G.L.C.); gianmarco.imperiali@edu.unife.it (G.I.); vittorio.orazi@edu.unife.it (V.O.); marco.tiseo@edu.unife.it (M.T.); anastasia.troia@edu.unife.it (A.T.); annalisa.rosso@unife.it (A.R.); 2 Department of Medical and Surgical Sciences, School of Public Health, University of Bologna, Via San Giacomo 12, 40138 Bologna, Italy; alessandro.bianconi4@studio.unibo.it (A.B.); matteo.fiore7@studio.unibo.it (M.F.)

**Keywords:** dengue fever, dengue vaccine, TAK-003, immunogenicity, vaccine efficacy, vaccine safety, meta-analysis

## Abstract

The TAK-003 dengue vaccine was licensed in Europe in December 2022, and the official recommendations from most EU countries are still under formulation. To support policymakers, we performed a meta-analysis to quantify TAK-003’s immunogenicity, efficacy and safety among seronegative and seropositive populations after the administration of one or two vaccine doses. We included trials retrieved from MEDLINE, Scopus and ClinicalTrials.gov. The outcomes were the rates of seroconversion, virologically confirmed dengue fever and serious adverse events after each vaccine dose. Data were combined using random-effect proportion or head-to-head meta-analyses. We retrieved a total of 19 datasets, including >20,000 participants. TAK-003 showed an excellent safety profile, and the immunogenicity after two doses against the four DENV serotypes was ≥90% among both adults and children/adolescents who were either seronegative or seropositive at baseline. A single dose was able to elicit a high immunogenic response among adults (≥70%) and children/adolescents (≥90%). The primary two-dose immunization course halved the risk of all types of virologically confirmed dengue fever among seropositive children/adolescents, but seronegative minors were only protected against the diseases caused by DENV-1 and DENV-2. Overall, the results support the use of TAK-003 for the prevention of dengue fever in the pediatric population of endemic countries. Uncertainties remain on the use of a single vaccine dose in non-endemic countries.

## 1. Introduction

Dengue fever, caused by an RNA *Flavivirus* named dengue virus (DENV) and primarily transmitted to humans by female *Aedes aegypti* mosquitoes [1,2], is the most common mosquito-borne viral disease, with an estimated 390 million people infected worldwide each year [3]. Of these, around 30% are symptomatic cases, and almost 1% fatal [4]. The infection is currently endemic in the tropical and subtropical regions of more than 100 countries [3], but an increasing dengue incidence in non-endemic areas is also being documented and is mainly due to the expansion of the *Aedes* mosquito vectors [4,5]. Additionally, dengue fever is increasingly contracted by travelers from non-endemic regions, with annual estimates ranging from 50 to 160 new cases/1000 individuals, depending on the epidemic year [6].

No therapeutic agents against dengue fever exist; therefore, in addition to vector control, preventive strategies inevitably rely on vaccination [7,8]. DENV has four distinct serotypes, each with specific antigenic characteristics; as a result, the need for a tetravalent formulation inducing homogeneous protection against all four serotypes has hindered the development of a specific vaccine [3]. Currently, only two vaccines are commercialized: CYD-TDV (Dengvaxia^®^), licensed in 2016, and TAK-003 (Qdenga^®^), approved by the European Medicines Agency in December 2022 [9]. CYD-TDV is currently recommended in 20 countries in a three-dose schedule only for subjects aged 9 to 45 years with laboratory-confirmed evidence of a past DENV infection, as seronegative individuals show a higher risk of severe disease following vaccination [3,4]. TAK-003 is intended to be administered in a two-dose schedule, three months apart, to individuals ≥ 4 years of age, regardless of their serological status [9], but the official recommendations from most EU countries are still under formulation, and a few questions are open [3,8]. In particular, TAK-003 is currently not recommended for those who cannot get two priming doses before leaving, as there is still uncertainty on the lowest number of doses that induce a satisfactory immune response [10]. Additionally, the evidence for TAK-003 relies on several phase I–III trials [1,4,11,12,13,14,15,16,17,18,19,20,21,22,23], with the main aim of assessing vaccine immunogenicity, while the evidence on its clinical efficacy and safety is highly fragmented and heterogeneous, and the results are complex to interpret by examining single studies. Therefore, we performed a meta-analysis in order to address these remaining questions and systematically appraise the evidence on the immunogenicity, clinical efficacy and safety of TAK-003 after the administration of a single dose or the complete two-dose schedule.

## 2. Materials and Methods

### 2.1. Search Strategy, Selection Criteria and Methodological Quality

We included clinical trials (either randomized or single-arm) evaluating the immunogenicity and/or the clinical efficacy and/or the safety of a live-attenuated, tetravalent dengue vaccine (TAK-003) among subjects of all ages. Two groups of investigators (A.B. and M.F.; and A.T., G.L.C., G.C., G.I., M.T. and V.O.) independently searched MEDLINE, Scopus and ClinicalTrials.gov using various combinations of the following search terms: “dengue OR dengue virus OR DENV” AND “vaccin*” AND “random*” (last search update 30 May 2024). While maintaining a common overall architecture, several alternative strings were used after adjustment for each database [24]. The reference lists of reviews and retrieved articles were also searched, and no language or date restrictions were used. The list of articles excluded after the full-text screening process and the reasons for the exclusion are reported in Table S1 online. The methodological quality of the included studies was assessed using the revised Cochrane risk-of-bias tool [25]. Discrepancies in the study selection and/or quality assessment were solved by a senior author (L.M.).

### 2.2. Primary Outcome: Immunogenicity

TAK-003 is a tetravalent vaccine based on DENV-2, one of the four co-circulating virus serotypes (DENV-1, DENV-2, DENV-3 and DENV-4), which was used as a laboratory-attenuated, backbone virus to generate the vaccine [26]. Recombinant viral serotypes 1, 3 and 4 were created by the substitution of the pre-membrane and envelope genes of the DENV-2 strain with those of the corresponding serotypes [21]. In accordance with the WHO guidelines [27], the detection of neutralizing antibodies against each of the four viral strains was performed using a 50% immuno-focus-reduction neutralization test (FRNT50), with antibody titers corresponding to the dilution, resulting in a ≥50% plaque reduction [1]. The main outcome of immunogenicity was seroconversion, defined as the proportion of subjects who were either seropositive before vaccination and achieved a four-fold or greater increase in antibody titer pre- to post-vaccination or who were seronegative and had a post-vaccination titer ≥ 1:4 [1]. Seroconversion was evaluated at two different time points (1 month after the first dose and 1 month after the second dose), which were chosen based on current immunization schedules, establishing a two-dose regimen, 90 days apart [9]. The control group included the subjects receiving either a placebo alone or in combination with other vaccines (the 9-valent anti-Human Papillomavirus vaccine 9vHPV; the inactivated anti-Hepatitis A-HAV Havrix 1440 vaccine; and the live, attenuated anti-Yellow Fever vaccine YF-17D).

### 2.3. Secondary Outcomes: Clinical Efficacy and Serious Adverse Events

Vaccine efficacy was evaluated from 30 days following a 2-dose primary immunization course (until the end of follow-up) against virologically confirmed dengue fever, defined as a febrile illness (≥38 °C) or illness clinically suspected to be dengue plus RT-PCR confirmation. To assess vaccine safety, we considered only serious adverse events (SAEs), defined as life-threatening events or events resulting in persistent disability, hospital admission or death and coded according to the Medical Dictionary for Regulatory Activities [4]. SAEs were considered either related or unrelated to the study vaccines by the investigators and were evaluated from the first day following dose 1 up to the end of follow-up.

### 2.4. Data Analysis

We first performed meta-analyses of proportions, combining the seroconversion rates of vaccinated individuals, irrespective of their baseline serological status, and against all four serotypes (a) 1 month after dose 1 and (b) 1 month after dose 2. All immunogenicity analyses were performed using Per-Protocol (PP) data and were stratified by vaccine strain (DENV-1, DENV-2, DENV-3 and DENV-4), serological status at baseline (only seropositive, only seronegative or mixed) and age class (children/adolescents and adults). Secondly, we compared the clinical efficacy of two doses of TAK-003 vaccine versus a placebo using random-effect head-to-head meta-analyses [28]. As for the proportion meta-analyses, head-to-head comparisons were run separately by vaccine strain, baseline serological status and age class and were performed using Intention-To-Treat (ITT) data. The results were expressed as risk ratios (RRs) and 95% confidence intervals (CIs), and the statistical heterogeneity was quantified using the I2 metric [29]. Head-to-head meta-analyses were also used to evaluate SAEs using the approach described above. We used Stata, version 13.1 (Stata Corp., College Station, TX, USA, 2013) and RevMan 5.4 (Copenhagen: The Nordic Cochrane Centre, The Cochrane Collaboration, 2020) to perform proportion and head-to-head meta-analyses, respectively.

## 3. Results

### 3.1. Characteristics of Included Studies

**Figure 1:**
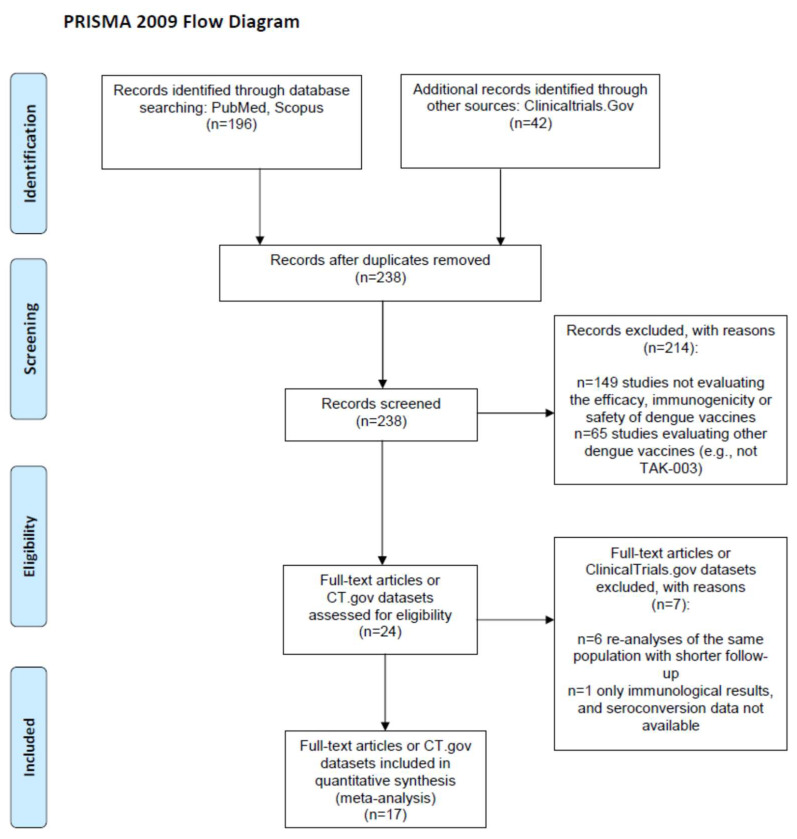
PRISMA 2009 flow diagram.

From the 238 screened records (Figure 1), we included 3 ClinicalTrials.gov reports [15,17,30] and 15 papers [1,4,11,12,13,14,16,18,19,20,21,22,23,31,32] reporting on 15 randomized clinical trials (RCTs) [1,4,11,12,13,14,16,17,18,19,21,22,23,30,31,32] and 2 single-arm trials [15,20] evaluating the immunogenicity and/or the safety and/or the efficacy of TAK-003 among children or adolescents (age at enrolment: 2–18 years; n = 7 [1,4,14,15,16,17,18,19]) or adults (18–60 years; n = 12 [11,12,13,20,21,22,23,30,31,32]). Two of the fifteen RCTs [31,32] did not compare vaccinated subjects versus controls (e.g., vaccines were compared at different doses); thus, they were included as single-arm trials in immunogenicity meta-analyses only. Immunogenicity [18] and efficacy or safety data [4] from a single RCT (NCT02747927) were extracted separately from two papers, and two studies reported the data separately for minors and adults [14,19] and were accordingly split into four datasets, leading to a total of nineteen datasets.

The main characteristics and the methodological quality of the included studies are reported in Table 1 and Table 2, respectively. Five studies were carried out in the USA, one in the UK, and all others were located in Asian or Central/South American countries, where dengue fever is endemic. All trials but one were funded by the vaccine manufacturer, and there was high homogeneity in the vaccine-dosing schedules (two doses administered 3 months apart, defined as the primary immunization course), dosage and administration (0.5 mL, subcutaneously), and comparator (most frequently, a placebo solution). The nineteen included datasets variously reported data on three outcomes, two vaccine doses, two age classes, four viral serotypes and three baseline serological statuses of the sample (mixed and seronegative or seropositive only), with a fragmentation of the results that led to a high number of stratified proportion or head-to-head meta-analyses, each including no more than nine datasets (Table 3).

### 3.2. Study Quality

The methodological quality of the 15 included RCTs is reported in Table 2. With the exception of the two RCTs that were included as single-arm trials in immunogenicity meta-analyses only [31,32], all the RCTs showed a low risk of overall risk of bias, as they carried a low risk of bias in each of the items of the Revised Cochrane risk-of-bias tool [25]: randomization process, deviation from intended interventions, missing outcome data, measurement of the outcome and selection of the reported results.

### 3.3. Immunogenicity 30 Days after the First Dose

Seventeen datasets (six pediatric [1,14,15,16,17,18] and eleven adult [11,12,13,14,19,20,21,22,23,30,31,32]) reported immunogenicity data after one [1,11,12,13,14,15,18,21,22,31,32] and/or two doses [1,11,12,13,14,15,16,17,18,20,21,22,23,30,32] (Table 3). The complete results extracted from single trials for each proportion meta-analysis, including more than one study, are shown in the Supplementary Materials (Tables S2–S62).

Overall, six datasets (n = 1533) were included in the meta-analyses evaluating the immunogenicity of the TAK-003 vaccine against each of the four viral serotypes one month after the primary dose among seronegative subjects [11,13,18,21,22,31] (Table 3 and Tables S3–S7). In the overall PP analysis, the pooled seroconversion rates slightly varied by viral serotype and were high for DENV-4 (78.5%) and DENV-3 (82.5%) and very high for DENV-1 and DENV-2 (>90%). In the four studies that examined vaccine protection against all serotypes, the pooled seroconversion rate after a single dose was 85.2% (95% CI: 67.4–97.0%). When the analyses were performed separately among adults and children/adolescents (Tables S2 and S8–S13), the seroconversion rates after the first dose were ≥91.0% for all serotypes among children/adolescents, while they ranged between 73.5% (DENV-4) and 90.9% (DENV-2) among adults.

In the two available datasets, including 1990 individuals [18,31], approximately all of the seropositive subjects showed protection against each of the serotypes after a single vaccine dose (Table 3 and Tables S14–S18). These results were largely influenced by the large trial on children/adolescents, while the single small trial on seropositive adults reported slightly lower seroconversion rates (82.8–94.3%, Tables S2 and S19).

A total of seven datasets (n = 3592) reported immunogenicity data from mixed samples (seropositive and seronegative participants or serological status not assessed; Table 3 and Tables S20–S24) [12,14,15,18,31,32]. The pooled seroconversion rates were below 90% only for the vaccine strain DENV-4 (83.7%) and were highest against the serotype DENV-2 (97.8%). A similar pattern was observed among adults and children/adolescents, with the lowest seroconversion rates for the DENV-1 serotype (80.5% and 89.6%, respectively; Tables S2 and S25–S33).

### 3.4. Immunogenicity 30 Days after the Second Dose

Six datasets evaluated immunogenicity one month after the primary course among seronegative subjects against each of the four viral serotypes (Table 3 and Tables S34–S38) [11,16,18,21,22,30]. Overall, the seroconversion rates increased after the second dose and were ≥98.9% for each viral strain. In the six trials that evaluated vaccine protection against all serotypes, the pooled seroconversion rate after two doses was 91.0% (95% CI: 82.2–97.2%). In both the pediatric and adult studies, the immunogenicity among seronegative subjects was >97% against each of the four tested strains (Tables S2 and S39–S48).

Only one trial assessed the immunogenicity after two doses among 1816 seropositive individuals (only minors), and it reported 100% protection against each viral serotype [18] (Table 3 and Table S2).

A total of eight datasets (n = 3789) reported immunogenicity data separately for each serotype after two doses from mixed samples [12,14,15,17,18,20,32], with pooled seroconversion rates above 96% for all viral strains (Table 3 and Tables S49–S53). Finally, very high protection (93.7%) was also observed in the four trials that considered the immunogenicity against all serotypes [1,14,15,19], and when the analyses were stratified by age, the lowest seroconversion rate was 89.7% (among the adults against the DENV-4 serotype; Tables S2 and S54–S62).

### 3.5. Efficacy

Three trials (four datasets) directly compared the efficacy of two doses of TAK-003 vaccine versus a placebo against virologically confirmed dengue fever [1,4,19]; only one of these datasets included adults [19], and only one trial reported the rates of diseases stratified by baseline serological status [4] (Table 4 and Figures S2 and S3). While the latter pediatric study had a very large sample (n = 20,067), the single trial on adults was seriously underpowered (n = 74) and could only be included in one meta-analysis, the results of which did not change. Therefore, all the following results on vaccine efficacy based on ITT analyses can be referred to children/adolescents only.

Overall, after the primary immunization course among both seropositive and seronegative subjects, vaccination significantly reduced the risk of a disease caused by the DENV-2 and DENV-1 serotypes by more than 80% and 40%, respectively (*p* < 0.001; Table 4; Figure S3). For the other two serotypes (DENV-3 and DENV-4), the results varied widely according to the baseline serological status: while the vaccine was able to significantly reduce the likelihood of dengue fever among seropositive subjects (RR = 0.50 for DENV-3; RR = 0.30 for DENV-4; both *p* < 0.001), seronegative children/adolescents were not protected (*p* > 0.20 for both serotypes; Table 4; Figure S3).

### 3.6. Safety–Adverse Events

The rates of all serious adverse events (SAEs) after the primary immunization course were reported separately by the vaccine arm in nine datasets [1,4,12,16,17,19,22,23], and the rates of product-related SAEs were available from six datasets [4,16,19,22,23] (Table 4; Figures S3 and S4). In most trials, the SAEs were recorded during the first 28 days after each dose, although a few studies reported data at longer time points [17,19,23].

In head-to-head meta-analyses based on ITT data, two doses of TAK-003 (compared to a placebo [1,4,12,16,19,22] or control vaccines [17,22]) did not increase significantly the risk of SAEs among adults [1,12,19,22] or children/adolescents [1,4,16,17,19] (both *p* > 0.05; Table 4; Figure S3).

The overall number of SAEs considered by the investigators to be related to the vaccine or placebo was one among the 15,017 subjects who received two vaccine doses and four among the 7448 controls. Two had hypersensitivity, two received a diagnosis of dengue, and one had dengue hemorrhagic fever [33]. No deaths were considered as potentially product-related. As for all SAEs, no significant association was found between vaccination and product-related SAEs among both adults and children or adolescents (Table 4; Figure S4).

### 3.7. Safety Small-Study Effects (Publication Bias)

As all meta-analyses included less than 10 studies, publication bias could not be assessed using funnel plots or formally tested through the Egger regression asymmetry test. In these cases, the available tests for publication are at very high risk of bias because of the lack of statistical power [34].

## 4. Discussion

The main findings of this meta-analysis are the following: (a) two doses of the TAK-003 vaccine induced the production of neutralizing antibodies against the four DENV serotypes in 90% or more of the vaccinated subjects, both adults and children/adolescents, who were seronegative or seropositive at baseline; (b) a single dose of vaccine was still able to elicit a high-to-very-high immunogenic response toward all serotypes among adults (≥70%) and children/adolescents (≥90%), with the lowest seroconversion rates observed among seronegative adults against the DENV-3 and DENV-4 serotypes (70–80%); (c) the primary two-dose immunization course reduced the risk of all types of virologically confirmed dengue fever among seropositive children/adolescents by more than 50%, but the efficacy widely varied by serotype among seronegative children, who were protected only against the diseases caused by DENV-1 and DENV-2; and (d) both one or two doses of the vaccine showed an excellent safety profile, with a frequency of serious adverse events that was comparable with those observed among the controls.

A previously tested tetravalent dengue vaccine (CYD-TDV) raised some safety concerns after the report of a higher risk of severe disease following a subsequent dengue infection in seronegative vaccinated subjects [35], and precautionary indications limited the use of this vaccine [36]. In this meta-analysis, TAK-003 showed a satisfactory safety profile, consistent with another dengue vaccine [37] and similar to other live-attenuated vaccines [38,39], with no indication of disease enhancement in dengue-naive participants who had a subsequent infection after the vaccination course. Additionally, one of the included studies, with a very large sample, had a relatively long follow-up (4.5 years) and provided robust evidence of the long-term safety (and immunogenicity) of TAK-003 [4].

Although the vaccine was able to provide an excellent long-term seroresponse against all serotypes in both seronegative and seropositive populations of any age, TAK-003 did not show a reduction in virologically confirmed dengue fever caused by DENV-3 and DENV-4 in dengue-naive participants. However, the results were dominated by a very large trial, in which there was low circulation of serotypes 3 and 4, so the serotype-stratified efficacy in seronegative people against these serotypes remains uncertain. In any case, the vaccine was able to reduce the overall rate of dengue fever by more than half in both seronegative and seropositive children/adolescents. Therefore, considering the results on safety, immunogenicity and efficacy following the administration of two vaccine doses, TAK-003 may certainly represent a central tool for the prevention of dengue fever among children and adolescents in highly endemic countries or within-country areas [2]. As regards non-endemic countries, where most of the population is expected to be seronegative, the administration of two doses of TAK-003 is indicated for travelers visiting endemic areas, especially if they are seropositive. In addition, a single vaccine dose—which was found to elicit a marked seroresponse—could also be considered for the population in non-endemic areas where surveillance data indicate a high risk of an ongoing dengue breakthrough. Aligned with the results of the meta-analysis, the Strategic Advisory Group of Experts on Immunization (SAGE) of the WHO recommended that TAK-003 should be introduced for children aged 6 to 16 years in sub-national settings with high dengue transmission intensity, while it did not suggest mass immunization in settings with a low-to-moderate risk of dengue transmission [36]. Also, the UK Joint Committee on Vaccination and Immunization (JCVI) recommended dengue vaccination using TAK-003 for travelers with immunological signs of previous dengue infection [40]. Analogously, the Belgium Superior Health Council recommends a complete vaccination course for travelers visiting high-risk endemic countries and with a previous history of dengue fever [10]. Further research is clearly needed to provide evidence on the effectiveness of recommending a single dose rather than the complete vaccination course in non-endemic countries.

This meta-analysis has some limitations that must be considered in interpreting the results. First, all included studies were pre-market RCTs sponsored by the manufacturer, and it will thus be essential to evaluate the real-world effectiveness and safety of TAK-003 through independent, post-marketing studies. Second, we found very scarce efficacy data on adults, and no data on the elderly and subjects with pre-existing diseases (all the retrieved trials included only healthy children or adults). Thus, further research is strongly needed to support future vaccination campaigns in adults, especially the frail population, who may be at higher risk of severe outcomes following infection [41,42]. Third, we included studies with heterogeneous formulations of TAK-003, as the concentration of the four live attenuated viruses contained in each vaccine dose varied among trials. However, all formulations included were above the minimum commercial plaque-forming unit (PFU) thresholds approved by the authorizing agencies for this vaccine [43]. Finally, although all of the included trials showed a low risk of overall bias, the heterogeneity was low in most of the meta-analyses, and no serious imprecision or inconsistency was noted across the studies, some of the performed meta-analyses included a limited number of studies, with larger studies disproportionately weighting the pooled estimates in their favor.

## 5. Conclusions

The TAK-003 tetravalent dengue vaccine appears to be safe and effective in triggering a long-lasting anticorpal response in both adults and children or adolescents, and even a single vaccine dose was able to elicit a high-to-very-high immunogenic response toward all serotypes. The primary two-dose immunization course reduced the overall risk of virologically confirmed dengue fever in children and adolescents by more than 50%, although the efficacy against dengue fever caused by the DENV-3 and DENV-4 serotypes in seronegative individuals remains uncertain. TAK-003 represents a valid intervention for the prevention of dengue fever in the pediatric population of endemic countries and travelers from non-endemic nations. Further, preferably independent studies are needed to clarify the vaccine efficacy for adults against serotypes 3 and 4 and the potential use of a single vaccine dose in non-endemic countries.

## Figures and Tables

**Table 1:** Characteristics of the included studies.

Id	First Author	Journal	Year	Age Range in Years; Mean	% Females	Country	Design	Blinding	Intervention	Control(s)	Outcome(s) Extracted	Sponsor	ClinicalTrials.gov ID
1	Osorio [11]	*Lancet Infect* *Dis*	2014	18–45; 20.5	70.0	Colombia	RCT	Double	2d (0.5 mL) SC or 2d (0.1 mL) ID M0-M3	Placebo	I	Takeda	NCT01224639
2	George [12]	*J Infect Dis*	2015	18–45; 31.5	46.0	USA	RCT	Double	2d (0.1 mL) SC M0-M3	Placebo	I; S	NIAIDS + Takeda	NCT01110551
3	Rupp [13]	*Vaccine*	2015	19–43; 30.0	60.0	Puerto Rico	RCT	Double	2d (0.5 mL) SC M0-M3	Placebo	I	Takeda	NCT01511250
4	Sirivichayakul [14]	*J Infect Dis*	2016	1.5–45; 9.8	47.8	Multi- country	RCT	Double	2d (0.5 mL) SC M0-M3	Placebo	I	Takeda	NCT01511250
5	Tricou [31]	*Vaccine*	2020	21–45; 30.9	52.3	Singapore	RCT *	Double	1d 0.5 mL SC	No controls, high vs. low vaccine dose *	I	Takeda	NCT02425098
6	Turner [32]	*Hum Vaccin* *Immunother*	2020	18–49; 32.1	49.4	USA	RCT *	Double	2d (0.5 mL) SC M0-M3	Liquid vs. lyophilized vaccine *	I	Takeda	NCT02193087
7	NCT02948829 [15]	ClinicalTrials.gov	2020	4–16; 6.7	50.5	Panama, Philippines	Single- arm trial	Open label	2d (0.5 mL) SC M0-M3	No control	I	Takeda	NCT02948829
8	Tricou [1]	*Lancet*	2020	2–18; 7.3	49.0	Multi- country	RCT	Double	2d (0.5 mL) SC M0-M3	Placebo	I; E; S	Takeda	NCT02302066
9	Patel [20]	*Hum Vaccin* *Immunother*	2020	18–60; 40.3	50.5	USA	Single- arm trial	Open label	2d (0.5 mL) SC M0-M3	No control	I	Takeda	NCT03771963
10	NCT04313244 [17]	ClinicalTrials.gov	2021	9–15; 11.2	49.8	Thailand	RCT	Double	2d (0.5 mL) SC M0-M3	9vHPV	I; S	Takeda	NCT04313244
11	Biswal [16]	*Rev Panam* *Salud Publica*	2021	12–17; 14.3	56.8	Mexico	RCT	Double	2d (0.5 mL) SC M0-M3	Placebo	I; S	Takeda	NCT03341637
12	Sirivichayakul [19]	*J Infect Dis*	2022	1.5–45; 9.8	47.8	Multi- country	RCT	Double	2d (0.5 mL) SC M0-M3	Placebo	E; S	Takeda	NCT01511250
13	López-Medina [18] and	*J Infect Dis*	2022	4–16; 9.6	49.7	Multi- country	RCT	Double	2d (0.5 mL) SC M0-M3	Placebo	I	Takeda	NCT02747927
14	Tricou [4]	*Lancet Global* *Health*	2024								E; S		
15	Tricou (A) [22]	*Vaccine*	2023	18–60; 35.4	35.3	UK	RCT	Double	2d (0.5 mL) SC M0-M3	HAV vaccine + placebo	I; S	Takeda	NCT03525119
16	Tricou [21]	*PLos Negl* *Trp Dis*	2023	18–60; 40.0	56.7	USA	RCT	Double	2d (0.5 mL) SC M0-M3	YF-17D + placebo	I	Takeda	NCT03342898
17	Tricou (B) [23,30] **	*Vaccine*	2023	18–60; 41.4	53.8	USA	RCT	Double	2d (0.5 mL) SC M0-M3	Placebo	I; S	Takeda	NCT03423173

1d: single dose; 2d: 2 doses, either administered through subcutaneous (SC) or intradermal (ID) injection; M0: first dose administered at baseline; M3: second dose administered after 3 months. 9vHPV: 9-valent anti-Human Papillomavirus vaccine. HAV: inactivated anti-Hepatitis A-HAV Havrix 1440 vaccine. YF-17D: live, attenuated anti-Yellow Fever vaccine. NIAIDS: National Institute of Allergy and Infectious Diseases. * Although the trial was randomized, it did not compare vaccinated subjects versus controls; thus, it was included as a single-arm trial in immunogenicity meta-analyses only. ** Immunogenicity data by strain were unavailable in the published manuscript and were extracted from the corresponding ClinicalTrials.gov record. I: Immunogenicity assessment evaluated through seroconversion, defined as the proportion of subjects either (a) seronegative at baseline, becoming seropositive after vaccination or (b) seropositive at baseline with a ≥4-fold rise in neutralizing antibody titers. Seropositive patients were defined as those with reciprocal neutralizing titers ≥ 10 against any of the dengue virus serotypes. In accordance with WHO guidelines, neutralizing antibodies were measured using a micro-neutralization assay, with antibody titers corresponding to the dilution, resulting in a ≥50% plaque reduction [1]. E: clinical efficacy, evaluated considering virologically confirmed dengue, defined as the onset of febrile illness (≥38 °C) or illness clinically suspected to be dengue plus RT-PCR confirmation. S: vaccine safety, assessed considering the onset of serious adverse events either related or unrelated to vaccine administration.

**Table 2:** Risk of bias of the included RCTs * assessed using the revised Cochrane risk-of-bias tool for randomized trials.

Study ID	Randomization Process	Deviations from Intended Interventions	Missing Outcome Data	Measurement of the Outcome	Selection of Reported Results	Overall Bias
Osorio (2014) [11]	Low risk	Low risk	Low risk	Low risk	Low risk	Low risk
George (2015) [12]	Low risk	Low risk	Low risk	Low risk	Low risk	Low risk
Rupp (2015) [13]	Low risk	Low risk	Low risk	Low risk	Low risk	Low risk
Sirivichayakul (2016) [14]	Low risk	Low risk	Low risk	Low risk	Low risk	Low risk
Tricou Vac (2020) [31]	Low risk *	Low risk	Low risk	Low risk	Low risk	Low risk
Turner (2020) [32]	Low risk *	Low risk	Low risk	Low risk	Low risk	Low risk
Tricou Lan (2020) [1]	Low risk	Low risk	Low risk	Low risk	Low risk	Low risk
NCT04313244 (2021) [17]	Low risk	Low risk	Low risk	Low risk	Low risk	Low risk
Biswal (2021) [16]	Low risk	Low risk	Low risk	Low risk	Low risk	Low risk
Sirivichayakul (2022) [19]	Low risk	Low risk	Low risk	Low risk	Low risk	Low risk
López-Medina (2022) [18]	Low risk	Low risk	Low risk	Low risk	Low risk	Low risk
Tricou (A) (2023) [22]	Low risk	Low risk	Low risk	Low risk	Low risk	Low risk
Tricou Plos (2023) [21]	Low risk	Low risk	Low risk	Low risk	Low risk	Low risk
Tricou (B) (2023) [23]	Low risk	Low risk	Low risk	Low risk	Low risk	Low risk
Tricou (2024) [4]	Low risk	Low risk	Low risk	Low risk	Low risk	Low risk

* Although the trial was randomized, it did not compare vaccinated subjects versus controls; thus, it was included as a single-arm trial in immunogenicity meta-analyses only.

**Table 3:** Rates of seroconversion * following the administration of a tetravalent dengue vaccine (TAK-003), according to number of doses, viral strain and baseline serological status. Data from single studies have been combined using proportion meta-analysis (random-effect model, PP data).

	30 Days after the 1st Dose		30 Days after the 2nd Dose	
	N. Studies (Sample)	Seroconversion *, % (95% CI)	N. Studies (Sample)	Seroconversion *, % (95% CI)
All ages				
*Seronegative subjects only*				
-All serotypes	4 (741)	85.2 (67.4–97.0)	6 (1278)	91.0 (82.2–97.2)
-DENV-1	6 (1533)	91.7 (86.4–95.8)	6 (1932)	99.9 (98.9–100)
-DENV-2	6 (1533)	90.9 (81.0–97.6)	6 (1932)	99.8 (98.6–100)
-DENV-3	6 (1533)	82.5 (69.9–92.5)	6 (1932)	99.0 (96.6–100)
-DENV-4	6 (1533)	78.5 (68.2–87.3)	6 (1932)	98.8 (96.4–100)
*Seropositive subjects only*				
-All serotypes	2 (150)	90.3 (84.9–94.7)	1 (41)	97.6 (87.4–99.6)
-DENV-1	2 (1990)	99.9 (99.7–100)	1 (1816)	100 (99.8–100)
-DENV-2	2 (1990)	99.9 (99.6–100)	1 (1816)	100 (99.8–100)
-DENV-3	2 (1990)	100 (99.8–100)	1 (1816)	100 (99.8–100)
-DENV-4	2 (1990)	100 (99.8–100)	1 (1816)	100 (99.8–100)
*Mixed samples (seropositive and seronegative or serological status not assessed)*				
-All serotypes	4 (527)	81.2 (64.9–93.3)	4 (505)	93.7 (88.9–97.3)
-DENV-1	7 (3592)	96.1 (92.2–98.8)	8 (3789)	100 (99.1–100)
-DENV-2	7 (3592)	97.8 (94.0–99.9)	8 (3789)	100 (100–100)
-DENV-3	7 (3592)	93.0 (84.7–98.5)	8 (3789)	100 (99.5–100)
-DENV-4	7 (3592)	83.7 (70.7–93.7)	8 (3789)	96.7 (88.3–100)

CI = confidence interval. PP = Per-Protocol. * Seroconversion was defined as the proportion of subjects either (a) seronegative at baseline, becoming seropositive after vaccination or (b) seropositive at baseline with a ≥4-fold rise in neutralizing antibody titers. Seropositive patients were defined as those with reciprocal neutralizing titers ≥ 10 against any of the dengue virus serotypes. In accordance with WHO guidelines, neutralizing antibodies were measured using a micro-neutralization assay, with antibody titers corresponding to the dilution, resulting in a ≥50% plaque reduction [1].

**Table 4:** Results of head-to-head meta-analyses on the efficacy * and safety ** of 2 doses of dengue TAK-003 vaccine vs. controls ^†^, according to age, viral strain and baseline serological status (ITT data).

		Overall Sample			Minors (2–18 y)	
Vaccine vs. Control, 2 Doses	N. Studies (Sample)	RR (95% CI)	*p*	N. Studies (Sample)	RR (95% CI)	*p*
**Virologically confirmed dengue fever**						
*Seronegative subjects only*						
-All viral serotypes	1 (5546)	0.47 (0.38–0.59)	<0.001	1 (5546)	0.47 (0.38–0.59)	<0.001
-DENV-1	1 (5546)	0.56 (0.41–0.75)	<0.001	1 (5546)	0.56 (0.41–0.75)	<0.001
-DENV-2	1 (5546)	0.12 (0.07–0.21)	<0.001	1 (5546)	0.12 (0.07–0.21)	<0.001
-DENV-3	1 (5546)	1.11 (0.62–1.99)	0.73	1 (5546)	1.11 (0.62–1.99)	0.73
-DENV-4	1 (5546)	1.97 (0.56–6.98)	0.29	1 (5546)	1.97 (0.56–6.98)	0.29
*Seropositive subjects only*						
-All viral serotypes	1 (14,517)	0.38 (0.32–0.44)	<0.001	1 (14,517)	0.38 (0.32–0.44)	<0.001
-DENV-1	1 (14,517)	0.44 (0.35–0.56)	<0.001	1 (14,517)	0.44 (0.35–0.56)	<0.001
-DENV-2	1 (14,517)	0.20 (0.15–0.27)	<0.001	1 (14,517)	0.20 (0.15–0.27)	<0.001
-DENV-3	1 (14,517)	0.50 (0.38–0.66)	<0.001	1 (14,517)	0.50 (0.38–0.66)	<0.001
-DENV-4	1 (14,517)	0.30 (0.15–0.62)	<0.001	1 (14,517)	0.30 (0.15–0.62)	<0.001
*Mixed samples (seropositive and seronegative or serological status not assessed)*						
-All viral serotypes	4 (20,825)	0.41 (0.36–0.46)	<0.001	3 (20,751)	0.41 (0.36–0.46)	<0.001
-DENV-1	2 (20,464)	0.53 (0.33–0.85)	<0.001	2 (20,464)	0.53 (0.33–0.85)	<0.001
-DENV-2	2 (20,464)	0.18 (0.13–0.23)	<0.001	2 (20,464)	0.18 (0.13–0.23)	<0.001
-DENV-3	2 (20,461)	0.58 (0.45–0.74)	<0.001	2 (20,461)	0.58 (0.45–0.74)	<0.001
-DENV-4	2 (20,461)	0.54 (0.31–0.94)	<0.001	2 (20,461)	0.54 (0.31–0.94)	<0.001
**Product-related SAEs**						
-All serotypes	6 (22,465)	0.36 (0.12–1.06)	0.06	3 (20,873)	0.29 (0.07–1.26)	0.10
**Any SAEs**						
-All serotypes	9 (23,381)	1.15 (0.77–1.71)	0.50	5 (21,765)	1.01 (0.70–1.44)	0.97

RR = random-effect risk ratio; 95% CI = confidence intervals; SAEs = serious adverse events; ITT: Intention-To-Treat. * Virologically confirmed dengue: febrile illness (≥38 °C) or illness clinically suspected to be dengue plus RT-PCR confirmation. ** Serious adverse events, either considered related or unrelated to vaccine administration. ^†^ Controls were subjects either administered a placebo solution (0.5 mL of phosphate-buffered saline solution) or control vaccines (9-valent anti-Human Papillomavirus-9vHPV vaccine, inactivated anti-Hepatitis A-HAV Havrix 1440 vaccine, or live, attenuated anti-Yellow Fever vaccine, YF-17D).

## Data Availability

All data are available from the studies included in the meta-analysis.

## Supplementary Materials

The following supporting information can be downloaded at: https://www.mdpi.com/article/10.3390/vaccines12070770/s1, Table S1: List of articles excluded after the full-text screening process and reasons of exclusion; Table S2: Rates of seroconversion following the administration of a tetravalent dengue vaccine (TAK-003), stratified by age class and according to number of doses, viral strain and baseline serological status; Tables S3–S62: Proportion meta-analyses estimating the rates of seroconversion following the first or second dose of TAK-003 vaccine in seronegative, seropositive or mixed subjects of all ages or only adults or only children/adolescents for all serotypes or by each serotype separately; Figure S1: Risk of virologically confirmed dengue fever among vaccinated vs. control subjects in the overall sample and by age class; Figure S2: Risk of virologically confirmed dengue fever among vaccinated vs. control subjects stratified by DENV serotype; Figure S3: Risk of any serious adverse events (SAEs) among vaccinated vs. control subjects in the overall sample and by age class; Figure S4: Risk of product-related serious adverse events (SAEs) among vaccinated vs. control subjects in the overall sample and by age class. References [1,4,5,31,33,44,45,46,47] are cited in the Supplementary Materials.

## References

[1] Tricou V., Saez-Llorens X., Yu D., Rivera L., Jimeno J., Villarreal A.C., Dato E., Saldana de Suman O., Montenegro N., DeAntonio R. (2020). Safety and immunogenicity of a tetravalent dengue vaccine in children aged 2-17 years: A randomised, placebo-controlled, phase 2 trial. Lancet.

[2] Wilder-Smith A. (2024). TAK-003 dengue vaccine as a new tool to mitigate dengue in countries with a high disease burden. Lancet Glob. Health.

[3] Paz-Bailey G., Adams L.E., Deen J., Anderson K.B., Katzelnick L.C. (2024). Dengue. Lancet.

[4] Tricou V., Yu D., Reynales H., Biswal S., Saez-Llorens X., Sirivichayakul C., Lopez P., Borja-Tabora C., Bravo L., Kosalaraksa P. (2024). Long-term efficacy and safety of a tetravalent dengue vaccine (TAK-003): 4.5-year results from a phase 3, randomised, double-blind, placebo-controlled trial. Lancet Glob. Health.

[5] Saez-Llorens X., Tricou V., Yu D., Rivera L., Jimeno J., Villarreal A.C., Dato E., Mazara S., Vargas M., Brose M. (2018). Immunogenicity and safety of one versus two doses of tetravalent dengue vaccine in healthy children aged 2–17 years in Asia and Latin America: 18-month interim data from a phase 2, randomised, placebo-controlled study. Lancet Infect. Dis..

[6] Halstead S., Wilder-Smith A. (2019). Severe dengue in travellers: Pathogenesis, risk and clinical management. J. Travel Med..

[7] Malik S., Ahsan O., Mumtaz H., Tahir Khan M., Sah R., Waheed Y. (2023). Tracing down the Updates on Dengue Virus-Molecular Biology, Antivirals, and Vaccine Strategies. Vaccines.

[8] Principi N., Esposito S. (2024). Development of Vaccines against Emerging Mosquito-Vectored Arbovirus Infections. Vaccines.

[9] European Medicines Agency (2022). Dengue Tetravalent Vaccine (Live, Attenuated) Takeda-Opinion on Medicine for Use Outside EU.

[10] Belgium Superior Health Council (2023). Vaccination against Dengue.

[11] Osorio J.E., Velez I.D., Thomson C., Lopez L., Jimenez A., Haller A.A., Silengo S., Scott J., Boroughs K.L., Stovall J.L. (2014). Safety and immunogenicity of a recombinant live attenuated tetravalent dengue vaccine (DENVax) in flavivirus-naive healthy adults in Colombia: A randomised, placebo-controlled, phase 1 study. Lancet Infect. Dis..

[12] George S.L., Wong M.A., Dube T.J., Boroughs K.L., Stovall J.L., Luy B.E., Haller A.A., Osorio J.E., Eggemeyer L.M., Irby-Moore S. (2015). Safety and Immunogenicity of a Live Attenuated Tetravalent Dengue Vaccine Candidate in Flavivirus-Naive Adults: A Randomized, Double-Blinded Phase 1 Clinical Trial. J. Infect. Dis..

[13] Rupp R., Luckasen G.J., Kirstein J.L., Osorio J.E., Santangelo J.D., Raanan M., Smith M.K., Wallace D., Gordon G.S., Stinchcomb D.T. (2015). Safety and immunogenicity of different doses and schedules of a live attenuated tetravalent dengue vaccine (TDV) in healthy adults: A Phase 1b randomized study. Vaccine.

[14] Sirivichayakul C., Barranco-Santana E.A., Esquilin-Rivera I., Oh H.M., Raanan M., Sariol C.A., Shek L.P., Simasathien S., Smith M.K., Velez I.D. (2016). Safety and Immunogenicity of a Tetravalent Dengue Vaccine Candidate in Healthy Children and Adults in Dengue-Endemic Regions: A Randomized, Placebo-Controlled Phase 2 Study. J. Infect. Dis..

[15] (2017). Takeda NCT02948829. Safety and Immunogenicity of Takeda’s Tetravalent Dengue Vaccine (TDV) in Healthy Children. NCT02948829.

[16] Biswal S., Mendez Galvan J.F., Macias Parra M., Galan-Herrera J.F., Carrascal Rodriguez M.B., Rodriguez Bueno E.P., Brose M., Rauscher M., LeFevre I., Wallace D. (2021). Immunogenicity and safety of a tetravalent dengue vaccine in dengue-naive adolescents in Mexico City. Rev. Panam. Salud Publica.

[17] (2021). Takeda NCT04313244. Immunogenicity and Safety of Dengue Tetravalent Vaccine (TDV) and Recombinant 9-Valent Human Papillomavirus Vaccine (9vHPV) in Participants Aged ≥9 to <15 Years. NCT04313244.

[18] López-Medina E., Biswal S., Saez-Llorens X., Borja-Tabora C., Bravo L., Sirivichayakul C., Vargas L.M., Alera M.T., Velásquez H., Reynales H. (2022). Efficacy of a Dengue Vaccine Candidate (TAK-003) in Healthy Children and Adolescents 2 Years after Vaccination. J. Infect. Dis..

[19] Sirivichayakul C., Barranco-Santana E.A., Rivera I.E., Kilbury J., Raanan M., Borkowski A., Papadimitriou A., Wallace D. (2022). Long-term Safety and Immunogenicity of a Tetravalent Dengue Vaccine Candidate in Children and Adults: A Randomized, Placebo-Controlled, Phase 2 Study. J. Infect. Dis..

[20] Patel S.S., Winkle P., Faccin A., Nordio F., LeFevre I., Tsoukas C.G. (2023). An open-label, Phase 3 trial of TAK-003, a live attenuated dengue tetravalent vaccine, in healthy US adults: Immunogenicity and safety when administered during the second half of a 24-month shelf-life. Hum. Vaccines Immunother..

[21] Tricou V., Essink B., Ervin J.E., Turner M., Escudero I., Rauscher M., Brose M., Lefevre I., Borkowski A., Wallace D. (2023). Immunogenicity and safety of concomitant and sequential administration of yellow fever YF-17D vaccine and tetravalent dengue vaccine candidate TAK-003: A phase 3 randomized, controlled study. PLoS Neglected Trop. Dis..

[22] Tricou V., Eyre S., Ramjee M., Collini P., Mojares Z., Loeliger E., Mandaric S., Rauscher M., Brose M., Lefevre I. (2023). A randomized phase 3 trial of the immunogenicity and safety of coadministration of a live-attenuated tetravalent dengue vaccine (TAK-003) and an inactivated hepatitis a (HAV) virus vaccine in a dengue non-endemic country. Vaccine.

[23] Tricou V., Winkle P.J., Tharenos L.M., Rauscher M., Escudero I., Hoffman E., LeFevre I., Borkowski A., Wallace D. (2023). Consistency of immunogenicity in three consecutive lots of a tetravalent dengue vaccine candidate (TAK-003): A randomized placebo-controlled trial in US adults. Vaccine.

[24] Flacco M.E., Manzoli L., Rosso A., Marzuillo C., Bergamini M., Stefanati A., Cultrera R., Villari P., Ricciardi W., Ioannidis J.P.A. (2018). Immunogenicity and safety of the multicomponent meningococcal B vaccine (4CMenB) in children and adolescents: A systematic review and meta-analysis. Lancet Infect. Dis..

[25] Higgins J.P., Altman D.G., Gotzsche P.C., Juni P., Moher D., Oxman A.D., Savovic J., Schulz K.F., Weeks L., Sterne J.A. (2011). The Cochrane Collaboration’s tool for assessing risk of bias in randomised trials. BMJ.

[26] Torres-Flores J.M., Reyes-Sandoval A., Salazar M.I. (2022). Dengue Vaccines: An Update. BioDrugs.

[27] Roehrig J.T., Hombach J., Barrett A.D. (2008). Guidelines for Plaque-Reduction Neutralization Testing of Human Antibodies to Dengue Viruses. Viral Immunol..

[28] DerSimonian R., Laird N. (1986). Meta-analysis in clinical trials. Control. Clin. Trials.

[29] Higgins J.P., Thompson S.G., Deeks J.J., Altman D.G. (2003). Measuring inconsistency in meta-analyses. BMJ.

[30] (2018). Takeda NCT03423173. Lot-to-Lot Consistency of 3 Lots of Tetravalent Dengue Vaccine (TDV) in Non-Endemic Country(Ies) for Dengue. NCT03423173.

[31] Tricou V., Low J.G., Oh H.M., Leo Y.S., Kalimuddin S., Wijaya L., Pang J., Ling L.M., Lee T.H., Brose M. (2020). Safety and immunogenicity of a single dose of a tetravalent dengue vaccine with two different serotype-2 potencies in adults in Singapore: A phase 2, double-blind, randomised, controlled trial. Vaccine.

[32] Turner M., Papadimitriou A., Winkle P., Segall N., Levin M., Doust M., Johnson C., Lucksinger G., Fierro C., Pickrell P. (2020). Immunogenicity and safety of lyophilized and liquid dengue tetravalent vaccine candidate formulations in healthy adults: A randomized, phase 2 clinical trial. Hum. Vaccines Immunother..

[33] Biswal S., Borja-Tabora C., Martinez Vargas L., Velasquez H., Theresa Alera M., Sierra V., Johana Rodriguez-Arenales E., Yu D., Wickramasinghe V.P., Duarte Moreira E. (2020). Efficacy of a tetravalent dengue vaccine in healthy children aged 4-16 years: A randomised, placebo-controlled, phase 3 trial. Lancet.

[34] Higgins J.P.T., Green S. (2011). Cochrane Handbook for Systematic Reviews of Interventions.

[35] Sridhar S., Luedtke A., Langevin E., Zhu M., Bonaparte M., Machabert T., Savarino S., Zambrano B., Moureau A., Khromava A. (2018). Effect of Dengue Serostatus on Dengue Vaccine Safety and Efficacy. N. Engl. J. Med..

[36] World Health Organization (2019). Dengue vaccine: WHO position paper, September 2018-Recommendations. Vaccine.

[37] Kallas E.G., Precioso A.R., Palacios R., Thome B., Braga P.E., Vanni T., Campos L.M.A., Ferrari L., Mondini G., da Graca Salomao M. (2020). Safety and immunogenicity of the tetravalent, live-attenuated dengue vaccine Butantan-DV in adults in Brazil: A two-step, double-blind, randomised placebo-controlled phase 2 trial. Lancet Infect. Dis..

[38] Furuya-Kanamori L., Xu C., Doi S.A.R., Clark J., Wangdi K., Mills D.J., Lau C.L. (2021). Comparison of immunogenicity and safety of licensed Japanese encephalitis vaccines: A systematic review and network meta-analysis. Vaccine.

[39] da Costa V.G., Marques-Silva A.C., Floriano V.G., Moreli M.L. (2014). Safety, immunogenicity and efficacy of a recombinant tetravalent dengue vaccine: A meta-analysis of randomized trials. Vaccine.

[40] Joint Committee on Vaccination and Immunisation (JCVI) (2024). Qdenga^®^ Dengue Vaccine Guidance.

[41] Lin R.J., Lee T.H., Leo Y.S. (2017). Dengue in the elderly: A review. Expert Rev. Anti-Infect. Ther..

[42] Badawi A., Velummailum R., Ryoo S.G., Senthinathan A., Yaghoubi S., Vasileva D., Ostermeier E., Plishka M., Soosaipillai M., Arora P. (2018). Prevalence of chronic comorbidities in dengue fever and West Nile virus: A systematic review and meta-analysis. PLoS ONE.

[43] European Medicines Agency (2022). Qdenga: EPAR-Medicine Overview.

[44] Tricou V., Gottardo R., Egan M.A., Clement F., Leroux-Roels G., Sáez-Llorens X., Borkowski A., Wallace D., Dean H.J. (2022). Characterization of the cell-mediated immune response to Takeda’s live-attenuated tetravalent dengue vaccine in adolescents participating in a phase 2 randomized controlled trial conducted in a dengue-endemic setting. Vaccine.

[45] Rivera L., Biswal S., Sáez-Llorens X., Reynales H., López-Medina E., Borja-Tabora C., Bravo L., Sirivichayakul C., Kosalaraksa P., Martinez Vargas L. (2022). Three-year efficacy and safety of Takeda’s dengue vaccine candidate (TAK-003). Clin. Infect. Dis..

[46] Biswal S., Reynales H., Saez-Llorens X., Lopez P., Borja-Tabora C., Kosalaraksa P., Sirivichayakul C., Watanaveeradej V., Rivera L., Espinoza F. (2019). Efficacy of a tetravalent dengue vaccine in healthy children and adolescents. N. Engl. J. Med..

[47] Sáez-Llorens X., Tricou V., Yu D., Rivera L., Tuboi S., Garbes P., Borkowski A., Wallace D. (2017). Safety and immunogenicity of one versus two doses of Takeda’s tetravalent dengue vaccine in children in Asia and Latin America: Interim results from a phase 2, randomised, placebo-controlled study. Lancet Infect. Dis..

