# Supplementary material for: Immunogenicity, Safety and Efficacy of the Dengue Vaccine TAK-003: A Meta-Analysis
Source: Vaccines (Basel). 2024 Jul 13;12(7):770. doi: 10.3390/vaccines12070770 (PMC11281463; doi:10.3390/vaccines12070770)
Supplement: Supplementary file 1 [file vaccines-12-00770-s001.zip › vaccines-3073938-supplementary.pdf]

SUPPLEMENTARY MATERIAL

Table S1. List of articles excluded after the full-text screening process and reasons of exclusion.

Reference	Trial ID	Reason for exclusion
Sirivichayakul C, Biswal S, Saez-Llorens X, et al. Efficacy and Safety of a Tetravalent Dengue Vaccine (TAK-003) in Children With Prior Japanese Encephalitis or Yellow Fever Vaccination. *J Infect Dis*. Published online April 29, 2024. doi:10.1093/infdis/jiae222	NCT02747927	Same population of Tricou Lancet Glob Health 2024 [4] ; data stratified by previous anti-Yellow Fever immunization
Tricou V, Gottardo R, Egan MA, et al. Characterization of the cell-mediated immune response to Takeda's live-attenuated tetravalent dengue vaccine in adolescents participating in a phase 2 randomized controlled trial conducted in a dengue-endemic setting. *Vaccine*. 2022;40(8):1143-1151. doi:10.1016/j.vaccine.2022.01.016	NCT02302066	Same population of Tricou Lancet Glob Health 2024 [4] (preliminary analysis)
Rivera L, Biswal S, Sáez-Llorens X, et al. Three-year Efficacy and Safety of Takeda's Dengue Vaccine Candidate (TAK-003). *Clin Infect Dis*. 2022;75(1):107-117. doi:10.1093/cid/ciab864	NCT02747927	Same population of Tricou Lancet Glob Health 2024 [4], but shorter follow-up
Biswal S, Borja-Tabora C, Martinez Vargas L, et al. Efficacy of a tetravalent dengue vaccine in healthy children aged 4-16 years: a randomised, placebo-controlled, phase 3 trial [published correction appears in Lancet. 2020 Apr 4;395(10230):1114]. *Lancet*. 2020;395(10234):1423-1433. doi:10.1016/S0140-6736(20)30414-1	NCT02747927	Same population of Tricou Lancet Glob Health 2024 [4], but shorter follow-up
Biswal S, Reynales H, Saez-Llorens X, et al. Efficacy of a Tetravalent Dengue Vaccine in Healthy Children and Adolescents. *N Engl J Med*. 2019;381(21):2009-2019. doi:10.1056/NEJMoa1903869	NCT02747927	Same population of Tricou Lancet 2020 [31]; only immunological data reported (reactivity of T-cell response)
Sáez-Llorens X, Tricou V, Yu D, et al. Immunogenicity and safety of one versus two doses of tetravalent dengue vaccine in healthy children aged 2-17 years in Asia and Latin America: 18-month interim data from a phase 2, randomised, placebo-controlled study. *Lancet Infect Dis*. 2018;18(2):162-170. doi:10.1016/S1473-3099(17)30632-1	NCT02302066	Same population of Tricou Lancet 2020 [1], but shorter follow-up
Sáez-Llorens X, Tricou V, Yu D, et al. Safety and immunogenicity of one versus two doses of Takeda's tetravalent dengue vaccine in children in Asia and Latin America: interim results from a phase 2, randomised, placebo-controlled study. *Lancet Infect Dis*. 2017;17(6):615-625. doi:10.1016/S1473-3099(17)30166-4	NCT02302066	Same population of Tricou Lancet 2020 [1], but shorter follow-up

Table S2. Rates of seroconversion * following the administration of a tetravalent Dengue vaccine (TAK-003), stratified by age-class, and according to number of doses, viral strain and baseline serological status. Data from single studies have been combined using proportion meta-analysis (random-effect model, PP data).

	30 days after the 1st dose		30 days after the 2nd dose	
	**N. studies (Sample)**	**Seroconversion*, % (95% CI)**	**N. studies (Sample)**	**Seroconversion*, % (95% CI)**
**Children and adolescents**				
Seronegative subjects only				
- All serotypes	2 (128)	96.2 (92.9-99.1)	2 (285)	99.0 (97.3-100)
- DENV-1	1 (702)	94.0 (92.0-95.5)	2 (945)	100 (99.8-100)
- DENV-2	1 (702)	99.0 (98.0-99.5)	2 (945)	100 (99.8-100)
- DENV-3	1 (702)	96.0 (94.3-97.2)	2 (945)	100 (99.8-100)
- DENV-4	1 (702)	91.0 (88.7-92.9)	2 (945)	100 (99.7-100)
Seropositive subjects only				
- All serotypes	2 (150)	90.3 (84.9-94.7)	1 (41)	97.6 (87.4-99.6)
- DENV-1	1 (1816)	100 (99.8-100)	1 (1816)	100 (99.8-100)
- DENV-2	1 (1816)	100 (99.8-100)	1 (1816)	100 (99.8-100)
- DENV-3	1 (1816)	100 (99.8-100)	1 (1816)	100 (99.8-100)
- DENV-4	1 (1816)	100 (99.8-100)	1 (1816)	100 (99.8-100)
Mixed samples (seropositive and seronegative, or serological status not assessed)				
- All serotypes	3 (481)	84.2 (65.7-96.5)	3 (459)	95.2 (92.1-97.6)
- DENV-1	3 (2906)	98.8 (96.7-99.9)	4 (3141)	100 (99.8-100)
- DENV-2	3 (2906)	99.5 (98.5-100)	4 (3141)	100 (99.8-100)
- DENV-3	3 (2906)	95.0 (80.7-100)	4 (3141)	100 (99.8-100)
- DENV-4	3 (2906)	89.6 (70.6-99.4)	4 (3141)	99.6 (97.2-100)
**Adults**				
Seronegative subjects only				
- All serotypes	2 (613)	80.3 (77.1-83.4)	4 (993)	87.7 (74.2-96.9)
- DENV-1	5 (831)	87.7 (76.0-96.1)	4 (987)	99.1 (96.4-100)
- DENV-2	5 (831)	90.9 (81.0-97.6)	4 (987)	97.5 (94.2-99.6)
- DENV-3	5 (831)	78.2 (63.4-90.1)	4 (987)	98.4 (95.7-99.9)
- DENV-4	5 (831)	73.5 (57.9-86.7)	4 (987)	97.2 (93.0-99.7)
Seropositive subjects only				
- All serotypes	0 (0)	--	0 (0)	--
- DENV-1	1 (174)	85.6 (79.7-90.1)	0 (0)	--
- DENV-2	1 (174)	82.8 (76.5-87.6)	0 (0)	--
- DENV-3	1 (174)	94.3 (89.7-96.8)	0 (0)	--
- DENV-4	1 (174)	94.3 (89.7-96.8)	0 (0)	--
Mixed samples (seropositive and seronegative, or serological status not assessed)				
- All serotypes	1 (46)	69.6 (55.2-80.9)	1 (46)	84.8 (71.8-92.4)
- DENV-1	4 (686)	92.3 (87.4-96.3)	4 (648)	99.5 (96.9-100)
- DENV-2	4 (686)	94.4 (80.5-100)	4 (648)	100 (99.8-100)
- DENV-3	4 (686)	90.7 (83.0-96.5)	4 (648)	99.8 (98.9-100)
- DENV-4	4 (686)	80.5 (74.1-86.2)	4 (648)	89.7 (71.2-99.8)

CI = confidence interval. PP = Per-protocol. * Seroconversion was defined as the proportion of subjects either: (a) seronegative at baseline, becoming seropositive after vaccination, or (b) seropositive at baseline with a ≥4-fold rise in neutralizing antibody titers. Seropositive patients were defined as those with reciprocal neutralizing titre ≥10 against any of the dengue virus serotypes. In accordance with WHO guidelines, neutralizing antibodies were measured using a micro-neutralization assay, with antibody titres corresponding to the dilution resulting in a ≥50% plaques reduction [6].

Table S3. Proportion meta-analysis estimating the rates of seroconversion following the first dose of TAK-003 vaccine in seronegative subjects of all ages (adults and children/adolescents) - All serotypes.

Study	ES	[95% Conf. Interval]	
Tricou L 2020 (2020)	0.738	0.589	0.847
Takeda NCT02948829 (	1.000	0.957	1.000
Tricou V A 2023 (202	0.717	0.592	0.815
Tricou PLOS 2023 (20	0.810	0.775	0.841
Random pooled ES	0.852	0.674	0.970

```
  Heterogeneity chi^2 =     56.322 (d.f. = 3) p =      0.000
  I^2 (variation in ES attributable to heterogeneity) =    94.673%
  Estimate of between-study variance Tau^2 =      0.170

  Test of ES=0 : z=     10.392 p =      0.000
```

Table S4. Proportion meta-analysis estimating the rates of seroconversion following the first dose of TAK-003 vaccine in seronegative subjects of all ages (adults and children/adolescents) - DENV-1 serotype.

Study	ES	[95% Conf. Interval]	
Lopez-Medina (2022)	0.940	0.920	0.955
Osorio (2014)	0.895	0.686	0.971
Tricou V A 2023 (202	0.883	0.778	0.942
Tricou PLOS 2023 (20	0.933	0.909	0.951
Rupp (2015)	1.000	0.867	1.000
Tricou V 2020 (2020)	0.799	0.733	0.852
Random pooled ES	0.917	0.864	0.958

```
  Heterogeneity chi^2 =     33.191 (d.f. = 5) p =      0.000
  I^2 (variation in ES attributable to heterogeneity) =    84.936%
  Estimate of between-study variance Tau^2 =      0.028

  Test of ES=0 : z=     28.807 p =      0.000
```

Table S5. Proportion meta-analysis estimating the rates of seroconversion following the first dose of TAK-003 vaccine in seronegative subjects of all ages (adults and children/adolescents) - DENV-2 serotype.

Study	ES	[95% Conf. Interval]	
Lopez-Medina (2022)	0.990	0.980	0.995
Osorio (2014)	0.684	0.460	0.846
Tricou V A 2023 (202	0.917	0.819	0.964
Tricou PLOS 2023 (20	0.944	0.922	0.960
Rupp (2015)	0.960	0.805	0.993
Tricou V 2020 (2020)	0.770	0.702	0.826
Random pooled ES	0.909	0.810	0.976

```
  Heterogeneity chi^2 =    107.849 (d.f. = 5) p =      0.000
  I^2 (variation in ES attributable to heterogeneity) =    95.364%
  Estimate of between-study variance Tau^2 =      0.104

  Test of ES=0 : z=     16.688 p =      0.000
```

Table S6. Proportion meta-analysis estimating the rates of seroconversion following the first dose of TAK-003 vaccine in seronegative subjects of all ages (adults and children/adolescents) - DENV-3 serotype.

Study	ES	[95% Conf. Interval]	
Lopez-Medina (2022)	0.960	0.943	0.972
Osorio (2014)	0.579	0.363	0.769
Tricou V A 2023 (202	0.850	0.739	0.919
Tricou PLOS 2023 (20	0.890	0.861	0.913
Rupp (2015)	0.840	0.653	0.936
Tricou V 2020 (2020)	0.655	0.582	0.722
Random pooled ES	0.825	0.699	0.925

```
  Heterogeneity chi^2 =    120.781 (d.f. = 5) p =      0.000
  I^2 (variation in ES attributable to heterogeneity) =    95.860%
  Estimate of between-study variance Tau^2 =      0.117

  Test of ES=0 : z=     14.240 p =      0.000
```

Table S7. Proportion meta-analysis estimating the rates of seroconversion following the first dose of TAK-003 vaccine in seronegative subjects of all ages (adults and children/adolescents) - DENV-4 serotype.

Study	ES	[95% Conf. Interval]	
Lopez-Medina (2022)	0.910	0.887	0.929
Osorio (2014)	0.632	0.410	0.809
Tricou V A 2023 (202	0.867	0.758	0.931
Tricou PLOS 2023 (20	0.888	0.859	0.912
Rupp (2015)	0.360	0.202	0.555
Tricou V 2020 (2020)	0.741	0.672	0.801
Random pooled ES	0.785	0.682	0.873

```
  Heterogeneity chi^2 =     69.730 (d.f. = 5) p =      0.000
  I^2 (variation in ES attributable to heterogeneity) =    92.829%
  Estimate of between-study variance Tau^2 =      0.065

  Test of ES=0 : z=     17.458 p =      0.000
```

Table S8. Proportion meta-analysis estimating the rates of seroconversion following the first dose of TAK-003 vaccine in seronegative children/adolescents - All serotypes.

Study	ES	[95% Conf. Interval]	
Tricou L 2020 (2020)	0.738	0.589	0.847
Takeda NCT02948829 (	1.000	0.957	1.000
Random pooled ES	0.962	0.919	0.991

```
  Test of ES=0 : z=     29.294 p =      0.000
```

Table S9. Proportion meta-analysis estimating the rates of seroconversion following the first dose of TAK-003 vaccine in seronegative adults - All serotypes.

Study	ES	[95% Conf.	Interval]
Tricou V A 2023 (202	0.717	0.592	0.815
Tricou PLOS 2023 (20	0.810	0.775	0.841
Random pooled ES	0.803	0.771	0.834

Test of ES=0 : z= 52.537 p = 0.000

Table S10. Proportion meta-analysis estimating the rates of seroconversion following the first dose of TAK-003 vaccine in seronegative adults - DENV-1 serotype.

Study	ES	[95% Conf.	Interval]
Osorio (2014)	0.895	0.686	0.971
Tricou V A 2023 (202	0.883	0.778	0.942
Tricou PLOS 2023 (20	0.933	0.909	0.951
Rupp (2015)	1.000	0.867	1.000
Tricou V 2020 (2020)	0.799	0.733	0.852
Random pooled ES	0.910	0.827	0.970

Heterogeneity chi^2 = 27.406 (d.f. = 4) p = 0.000
I^2 (variation in ES attributable to heterogeneity) = 85.404%
Estimate of between-study variance Tau^2 = 0.055

Test of ES=0 : z= 19.361 p = 0.000

Table S11. Proportion meta-analysis estimating the rates of seroconversion following the first dose of TAK-003 vaccine in seronegative adults - DENV-2 serotype.

Study	ES	[95% Conf.	Interval]
Osorio (2014)	0.684	0.460	0.846
Tricou V A 2023 (202	0.917	0.819	0.964
Tricou PLOS 2023 (20	0.944	0.922	0.960
Rupp (2015)	0.960	0.805	0.993
Tricou V 2020 (2020)	0.770	0.702	0.826
Random pooled ES	0.877	0.760	0.961

Heterogeneity chi^2 = 43.670 (d.f. = 4) p = 0.000
I^2 (variation in ES attributable to heterogeneity) = 90.840%
Estimate of between-study variance Tau^2 = 0.094

Test of ES=0 : z= 14.926 p = 0.000

Table S12. Proportion meta-analysis estimating the rates of seroconversion following the first dose of TAK-003 vaccine in seronegative adults - DENV-3 serotype.

Study	ES	[95% Conf. Interval]	
Osorio (2014)	0.579	0.363	0.769
Tricou V A 2023 (202	0.850	0.739	0.919
Tricou PLOS 2023 (20	0.890	0.861	0.913
Rupp (2015)	0.840	0.653	0.936
Tricou V 2020 (2020)	0.655	0.582	0.722
Random pooled ES	0.782	0.634	0.901

```
Heterogeneity chi^2 =     51.216 (d.f. = 4) p =      0.000
I^2 (variation in ES attributable to heterogeneity) =    92.190%
Estimate of between-study variance Tau^2 =      0.111

Test of ES=0 : z=     12.340 p =      0.000
```

Table S13. Proportion meta-analysis estimating the rates of seroconversion following the first dose of TAK-003 vaccine in seronegative adults - DENV-4 serotype.

Study	ES	[95% Conf. Interval]	
Osorio (2014)	0.632	0.410	0.809
Tricou V A 2023 (202	0.867	0.758	0.931
Tricou PLOS 2023 (20	0.888	0.859	0.912
Rupp (2015)	0.360	0.202	0.555
Tricou V 2020 (2020)	0.741	0.672	0.801
Random pooled ES	0.735	0.579	0.867

```
Heterogeneity chi^2 =     52.726 (d.f. = 4) p =      0.000
I^2 (variation in ES attributable to heterogeneity) =    92.414%
Estimate of between-study variance Tau^2 =      0.115

Test of ES=0 : z=     11.524 p =      0.000
```

Table S14. Proportion meta-analysis estimating the rates of seroconversion following the first dose of TAK-003 vaccine in seropositive subjects of all ages (adults and children/adolescents) - All serotypes.

Study	ES	[95% Conf. Interval]	
Tricou L 2020 (2020)	0.854	0.716	0.931
Takeda NCT02948829 (	0.917	0.850	0.956
Random pooled ES	0.903	0.849	0.947

```
Test of ES=0 : z=     28.978 p =      0.000
```

Table S15. Proportion meta-analysis estimating the rates of seroconversion following the first dose of TAK-003 vaccine in seropositive subjects of all ages (adults and children/adolescents) – DENV-1 serotype.

Study	ES	[95% Conf.	Interval]
Lopez-Medina (2022)	1.000	0.998	1.000
Tricou V 2020 (2020)	0.856	0.797	0.901
Random pooled ES	0.999	0.997	1.000

Test of ES=0 : z= 133.660 p = 0.000

Table S16. Proportion meta-analysis estimating the rates of seroconversion following the first dose of TAK-003 vaccine in seropositive subjects of all ages (adults and children/adolescents) – DENV-2 serotype.

Study	ES	[95% Conf.	Interval]
Lopez-Medina (2022)	1.000	0.998	1.000
Tricou V 2020 (2020)	0.828	0.765	0.876
Random pooled ES	0.999	0.996	1.000

Test of ES=0 : z= 133.355 p = 0.000

Table S17. Proportion meta-analysis estimating the rates of seroconversion following the first dose of TAK-003 vaccine in seropositive subjects of all ages (adults and children/adolescents) – DENV-3 serotype.

Study	ES	[95% Conf.	Interval]
Lopez-Medina (2022)	1.000	0.998	1.000
Tricou V 2020 (2020)	0.943	0.897	0.968
Random pooled ES	1.000	0.998	1.000

Test of ES=0 : z= 134.788 p = 0.000

Table S18. Proportion meta-analysis estimating the rates of seroconversion following the first dose of TAK-003 vaccine in seropositive subjects of all ages (adults and children/adolescents) – DENV-4 serotype.

Study	ES	[95% Conf.	Interval]
Lopez-Medina (2022)	1.000	0.998	1.000
Tricou V 2020 (2020)	0.943	0.897	0.968
Random pooled ES	1.000	0.998	1.000

Test of ES=0 : z= 134.788 p = 0.000

Table S19. Proportion meta-analysis estimating the rates of seroconversion following the first dose of TAK-003 vaccine in seropositive children/adolescents - All serotypes.

```
          Study     |     ES    [95% Conf. Interval]
---------------------+---------------------------------------------------
Tricou L 2020 (2020) |     0.854      0.716        0.931
Takeda NCT02948829 ( |     0.917      0.850        0.956
---------------------+---------------------------------------------------
Random pooled  ES    |     0.903      0.849        0.947
---------------------+---------------------------------------------------

  Test of ES=0 : z=     28.978 p =      0.000
```

Table S20. Proportion meta-analysis estimating the rates of seroconversion following the first dose of TAK-003 vaccine in seropositive or seronegative (mixed) subjects of all ages (adults and children/adolescents) - All serotypes.

```
          Study     |     ES    [95% Conf. Interval]
---------------------+---------------------------------------------------
Tricou L 2020 (2020) |     0.795      0.696        0.868
Takeda NCT02948829 ( |     0.954      0.915        0.976
Sirivichayakul AD 20 |     0.696      0.552        0.809
Sirivichayakul CH 20 |     0.734      0.669        0.790
---------------------+---------------------------------------------------
Random pooled  ES    |     0.812      0.649        0.933
---------------------+---------------------------------------------------

  Heterogeneity chi^2 =     49.302 (d.f. = 3) p =      0.000
  I^2 (variation in ES attributable to heterogeneity) =    93.915%
  Estimate of between-study variance Tau^2 =      0.128

  Test of ES=0 : z=     11.443 p =      0.000
```

Table S21. Proportion meta-analysis estimating the rates of seroconversion following the first dose of TAK-003 vaccine in seropositive or seronegative (mixed) subjects of all ages (adults and children/adolescents) – DENV-1 serotype.

```
          Study     |     ES    [95% Conf. Interval]
---------------------+---------------------------------------------------
Lopez-Medina (2022)  |     0.983      0.978        0.988
Takeda NCT02948829 ( |     0.968      0.931        0.985
Turner (2020)        |     0.936      0.910        0.955
Sirivichayakul AD 20 |     0.935      0.825        0.978
Sirivichayakul CH 20 |     1.000      0.981        1.000
George (2015)        |     0.667      0.391        0.862
Tricou V 2020 (2020) |     0.914      0.863        0.947
---------------------+---------------------------------------------------
Random pooled  ES    |     0.961      0.922        0.988
---------------------+---------------------------------------------------

  Heterogeneity chi^2 =     66.792 (d.f. = 6) p =      0.000
  I^2 (variation in ES attributable to heterogeneity) =    91.017%
  Estimate of between-study variance Tau^2 =      0.035

  Test of ES=0 : z=     32.200 p =      0.000
```

Table S22. Proportion meta-analysis estimating the rates of seroconversion following the first dose of TAK-003 vaccine in seropositive or seronegative (mixed) subjects of all ages (adults and children/adolescents) – DENV-2 serotype.

Study	ES	[95% Conf. Interval]	
Lopez-Medina (2022)	0.997	0.994	0.999
Takeda NCT02948829 (	0.995	0.970	0.999
Turner (2020)	0.996	0.984	0.999
Sirivichayakul AD 20	0.935	0.825	0.978
Sirivichayakul CH 20	0.985	0.957	0.995
George (2015)	0.917	0.646	0.985
Tricou V 2020 (2020)	0.856	0.797	0.901
Random pooled ES	0.978	0.940	0.999

```
Heterogeneity chi^2 =     86.957 (d.f. = 6) p =      0.000
I^2 (variation in ES attributable to heterogeneity) =    93.100%
Estimate of between-study variance Tau^2 =      0.046
Test of ES=0 : z=     29.474 p =      0.000
```

Table S23. Proportion meta-analysis estimating the rates of seroconversion following the first dose of TAK-003 vaccine in seropositive or seronegative (mixed) subjects of all ages (adults and children/adolescents) – DENV-3 serotype.

Study	ES	[95% Conf. Interval]	
Lopez-Medina (2022)	0.989	0.984	0.992
Takeda NCT02948829 (	0.995	0.970	0.999
Turner (2020)	0.934	0.907	0.953
Sirivichayakul AD 20	0.935	0.825	0.978
Sirivichayakul CH 20	0.778	0.716	0.830
George (2015)	0.917	0.646	0.985
Tricou V 2020 (2020)	0.828	0.765	0.876
Random pooled ES	0.930	0.847	0.985

```
Heterogeneity chi^2 =    195.852 (d.f. = 6) p =      0.000
I^2 (variation in ES attributable to heterogeneity) =    96.936%
Estimate of between-study variance Tau^2 =      0.109
Test of ES=0 : z=     18.542 p =      0.000
```

Table S24. Proportion meta-analysis estimating the rates of seroconversion following the first dose of TAK-003 vaccine in seropositive or seronegative (mixed) subjects of all ages (adults and children/adolescents) – DENV-4 serotype.

Study	ES	[95% Conf. Interval]	
Lopez-Medina (2022)	0.975	0.968	0.980
Takeda NCT02948829 (	0.930	0.883	0.958
Turner (2020)	0.800	0.760	0.834
Sirivichayakul AD 20	0.717	0.575	0.827
Sirivichayakul CH 20	0.714	0.649	0.772
George (2015)	0.667	0.391	0.862
Tricou V 2020 (2020)	0.856	0.797	0.901
Random pooled ES	0.837	0.704	0.937

```
Heterogeneity chi^2 =    287.197 (d.f. = 6) p =      0.000
I^2 (variation in ES attributable to heterogeneity) =    97.911%
Estimate of between-study variance Tau^2 =      0.161
Test of ES=0 : z=     13.662 p =      0.000
```

Table S25. Proportion meta-analysis estimating the rates of seroconversion following the first dose of TAK-003 vaccine in seropositive or seronegative (mixed) children/adolescents - All serotypes.

Study	ES	[95% Conf. Interval]	
Tricou L 2020 (2020)	0.795	0.696	0.868
Takeda NCT02948829 (	0.954	0.915	0.976
Sirivichayakul CH 20	0.734	0.669	0.790
Random pooled ES	0.842	0.657	0.965

```
  Heterogeneity chi^2 =     43.318 (d.f. = 2) p =      0.000
  I^2 (variation in ES attributable to heterogeneity) =    95.383%
  Estimate of between-study variance Tau^2 =      0.136

  Test of ES=0 : z=     10.200 p =      0.000
```

Table S26. Proportion meta-analysis estimating the rates of seroconversion following the first dose of TAK-003 vaccine in seropositive or seronegative (mixed) children/adolescents – DENV-1 serotype.

Study	ES	[95% Conf. Interval]	
Lopez-Medina (2022)	0.983	0.978	0.988
Takeda NCT02948829 (	0.968	0.931	0.985
Sirivichayakul CH 20	1.000	0.981	1.000
Random pooled ES	0.988	0.967	0.999

```
  Heterogeneity chi^2 =      9.743 (d.f. = 2) p =      0.008
  I^2 (variation in ES attributable to heterogeneity) =    79.473%
  Estimate of between-study variance Tau^2 =      0.011

  Test of ES=0 : z=     41.284 p =      0.000
```

Table S27. Proportion meta-analysis estimating the rates of seroconversion following the first dose of TAK-003 vaccine in seropositive or seronegative (mixed) children/adolescents – DENV-2 serotype.

Study	ES	[95% Conf. Interval]	
Lopez-Medina (2022)	0.997	0.994	0.999
Takeda NCT02948829 (	0.995	0.970	0.999
Sirivichayakul CH 20	0.985	0.957	0.995
Random pooled ES	0.995	0.985	1.000

```
  Heterogeneity chi^2 =      4.962 (d.f. = 2) p =      0.084
  I^2 (variation in ES attributable to heterogeneity) =    59.696%
  Estimate of between-study variance Tau^2 =      0.004

  Test of ES=0 : z=     60.441 p =      0.000
```

Table S28. Proportion meta-analysis estimating the rates of seroconversion following the first dose of TAK-003 vaccine in seropositive or seronegative (mixed) children/adolescents – DENV-3 serotype.

Study	ES	[95% Conf. Interval]	
Lopez-Medina (2022)	0.989	0.984	0.992
Takeda NCT02948829 (	0.995	0.970	0.999
Sirivichayakul CH 20	0.778	0.716	0.830
Random pooled ES	0.950	0.807	1.000

```
  Heterogeneity chi^2 =    113.323 (d.f. = 2) p =      0.000
  I^2 (variation in ES attributable to heterogeneity) =    98.235%
  Estimate of between-study variance Tau^2 =      0.159

  Test of ES=0 : z=     11.279 p =      0.000
```

Table S29. Proportion meta-analysis estimating the rates of seroconversion following the first dose of TAK-003 vaccine in seropositive or seronegative (mixed) children/adolescents – DENV-4 serotype.

Study	ES	[95% Conf. Interval]	
Lopez-Medina (2022)	0.975	0.968	0.980
Takeda NCT02948829 (	0.930	0.883	0.958
Sirivichayakul CH 20	0.714	0.649	0.772
Random pooled ES	0.896	0.706	0.994

```
  Heterogeneity chi^2 =    128.716 (d.f. = 2) p =      0.000
  I^2 (variation in ES attributable to heterogeneity) =    98.446%
  Estimate of between-study variance Tau^2 =      0.181

  Test of ES=0 : z=      9.755 p =      0.000
```

Table S30. Proportion meta-analysis estimating the rates of seroconversion following the first dose of TAK-003 vaccine in seropositive or seronegative (mixed) adults – DENV-1 serotype.

Study	ES	[95% Conf. Interval]	
Turner (2020)	0.936	0.910	0.955
Sirivichayakul AD 20	0.935	0.825	0.978
George (2015)	0.667	0.391	0.862
Tricou V 2020 (2020)	0.914	0.863	0.947
Random pooled ES	0.923	0.874	0.963

```
  Heterogeneity chi^2 =      7.313 (d.f. = 3) p =      0.063
  I^2 (variation in ES attributable to heterogeneity) =    58.979%
  Estimate of between-study variance Tau^2 =      0.013

  Test of ES=0 : z=     30.173 p =      0.000
```

Table S31. Proportion meta-analysis estimating the rates of seroconversion following the first dose of TAK-003 vaccine in seropositive or seronegative (mixed) adults – DENV-2 serotype.

Study	ES	[95% Conf. Interval]	
Turner (2020)	0.996	0.984	0.999
Sirivichayakul AD 20	0.935	0.825	0.978
George (2015)	0.917	0.646	0.985
Tricou V 2020 (2020)	0.856	0.797	0.901
Random pooled ES	0.944	0.805	1.000

```
  Heterogeneity chi^2 =     54.644 (d.f. = 3) p =      0.000
  I^2 (variation in ES attributable to heterogeneity) =    94.510%
  Estimate of between-study variance Tau^2 =      0.152

  Test of ES=0 : z=     11.724 p =      0.000
```

Table S32. Proportion meta-analysis estimating the rates of seroconversion following the first dose of TAK-003 vaccine in seropositive or seronegative (mixed) adults – DENV-3 serotype.

Study	ES	[95% Conf. Interval]	
Turner (2020)	0.934	0.907	0.953
Sirivichayakul AD 20	0.935	0.825	0.978
George (2015)	0.917	0.646	0.985
Tricou V 2020 (2020)	0.828	0.765	0.876
Random pooled ES	0.907	0.830	0.965

```
  Heterogeneity chi^2 =     14.617 (d.f. = 3) p =      0.002
  I^2 (variation in ES attributable to heterogeneity) =    79.475%
  Estimate of between-study variance Tau^2 =      0.034

  Test of ES=0 : z=     20.666 p =      0.000
```

Table S33. Proportion meta-analysis estimating the rates of seroconversion following the first dose of TAK-003 vaccine in seropositive or seronegative (mixed) adults – DENV-4 serotype.

Study	ES	[95% Conf. Interval]	
Turner (2020)	0.800	0.760	0.834
Sirivichayakul AD 20	0.717	0.575	0.827
George (2015)	0.667	0.391	0.862
Tricou V 2020 (2020)	0.856	0.797	0.901
Random pooled ES	0.805	0.741	0.862

```
  Heterogeneity chi^2 =      6.759 (d.f. = 3) p =      0.080
  I^2 (variation in ES attributable to heterogeneity) =    55.612%
  Estimate of between-study variance Tau^2 =      0.011

  Test of ES=0 : z=     27.111 p =      0.000
```

Table S34. Proportion meta-analysis estimating the rates of seroconversion following the second dose of TAK-003 vaccine in seronegative subjects of all ages (adults and children/adolescents) - All serotypes.

Study	ES	[95% Conf. Interval]	
Tricou L 2020 (2020)	0.857	0.722	0.933
Tricou V A 2023 (202	0.909	0.804	0.961
Tricou PLOS 2023 (20	0.980	0.964	0.989
Rupp (2015)	0.360	0.202	0.555
Tricou V B 2023 (202	0.956	0.930	0.973
Biswal (2021)	0.996	0.977	0.999
Random pooled ES	0.910	0.822	0.972

```
Heterogeneity chi^2 =     82.985 (d.f. = 5) p =      0.000
I^2 (variation in ES attributable to heterogeneity) =    93.975%
Estimate of between-study variance Tau^2 =      0.087

Test of ES=0 : z=     18.457 p =      0.000
```

Table S35. Proportion meta-analysis estimating the rates of seroconversion following the second dose of TAK-003 vaccine in seronegative subjects of all ages (adults and children/adolescents) – DENV-1 serotype.

Study	ES	[95% Conf. Interval]	
Lopez-Medina (2022)	1.000	0.995	1.000
Osorio (2014)	0.947	0.754	0.991
Tricou V A 2023 (202	1.000	0.935	1.000
Tricou PLOS 2023 (20	0.996	0.987	0.999
Biswal (2021)	1.000	0.984	1.000
Takeda NCT03423173 (	0.978	0.958	0.989
Random pooled ES	0.999	0.989	1.000

```
Heterogeneity chi^2 =     21.688 (d.f. = 5) p =      0.001
I^2 (variation in ES attributable to heterogeneity) =    76.945%
Estimate of between-study variance Tau^2 =      0.012

Test of ES=0 : z=     52.305 p =      0.000
```

Table S36. Proportion meta-analysis estimating the rates of seroconversion following the second dose of TAK-003 vaccine in seronegative subjects of all ages (adults and children/adolescents) – DENV-2 serotype.

Study	ES	[95% Conf. Interval]	
Lopez-Medina (2022)	1.000	0.995	1.000
Osorio (2014)	0.789	0.567	0.915
Tricou V A 2023 (202	1.000	0.935	1.000
Tricou PLOS 2023 (20	0.993	0.981	0.997
Biswal (2021)	1.000	0.984	1.000
Takeda NCT03423173 (	0.992	0.976	0.997
Random pooled ES	0.998	0.986	1.000

```
Heterogeneity chi^2 =     24.285 (d.f. = 5) p =      0.000
I^2 (variation in ES attributable to heterogeneity) =    79.411%
Estimate of between-study variance Tau^2 =      0.014

Test of ES=0 : z=     49.285 p =      0.000
```

Table S37. Proportion meta-analysis estimating the rates of seroconversion following the second dose of TAK-003 vaccine in seronegative subjects of all ages (adults and children/adolescents) – DENV-3 serotype.

Study	ES	[95% Conf. Interval]	
Lopez-Medina (2022)	1.000	0.995	1.000
Osorio (2014)	0.947	0.754	0.991
Tricou V A 2023 (202	0.927	0.827	0.971
Tricou PLOS 2023 (20	0.989	0.976	0.995
Biswal (2021)	1.000	0.984	1.000
Takeda NCT03423173 (	0.967	0.944	0.981
Random pooled ES	0.990	0.966	1.000

Heterogeneity chi^2 = 42.864 (d.f. = 5) p = 0.000
I^2 (variation in ES attributable to heterogeneity) = 88.335%
Estimate of between-study variance Tau^2 = 0.027

Test of ES=0 : z= 36.455 p = 0.000

Table S38. Proportion meta-analysis estimating the rates of seroconversion following the second dose of TAK-003 vaccine in seronegative subjects of all ages (adults and children/adolescents) – DENV-4 serotype.

Study	ES	[95% Conf. Interval]	
Lopez-Medina (2022)	1.000	0.995	1.000
Osorio (2014)	0.737	0.512	0.882
Tricou V A 2023 (202	0.964	0.877	0.990
Tricou PLOS 2023 (20	0.987	0.974	0.994
Biswal (2021)	0.996	0.977	0.999
Takeda NCT03423173 (	0.984	0.965	0.992
Random pooled ES	0.988	0.964	1.000

Heterogeneity chi^2 = 38.578 (d.f. = 5) p = 0.000
I^2 (variation in ES attributable to heterogeneity) = 87.039%
Estimate of between-study variance Tau^2 = 0.024

Test of ES=0 : z= 38.081 p = 0.000

Table S39. Proportion meta-analysis estimating the rates of seroconversion following the second dose of TAK-003 vaccine in seronegative children/adolescents - All serotypes.

Study	ES	[95% Conf. Interval]	
Tricou L 2020 (2020)	0.857	0.722	0.933
Biswal (2021)	0.996	0.977	0.999
Random pooled ES	0.990	0.973	1.000

Test of ES=0 : z= 46.907 p = 0.000

Table S40. Proportion meta-analysis estimating the rates of seroconversion following the second dose of TAK-003 vaccine in seronegative children/adolescents – DENV-1 serotype.

Study	ES	[95% Conf. Interval]	
Lopez-Medina (2022)	1.000	0.995	1.000
Biswal (2021)	1.000	0.984	1.000
Random pooled ES	1.000	0.998	1.000

Test of ES=0 : z= 93.640 p = 0.000

Table S41. Proportion meta-analysis estimating the rates of seroconversion following the second dose of TAK-003 vaccine in seronegative children/adolescents – DENV-2 serotype.

Study	ES	[95% Conf. Interval]	
Lopez-Medina (2022)	1.000	0.995	1.000
Biswal (2021)	1.000	0.984	1.000
Random pooled ES	1.000	0.998	1.000

Test of ES=0 : z= 93.640 p = 0.000

Table S42. Proportion meta-analysis estimating the rates of seroconversion following the second dose of TAK-003 vaccine in seronegative children/adolescents – DENV-3 serotype.

Study	ES	[95% Conf. Interval]	
Lopez-Medina (2022)	1.000	0.995	1.000
Biswal (2021)	1.000	0.984	1.000
Random pooled ES	1.000	0.998	1.000

Test of ES=0 : z= 93.640 p = 0.000

Table S43. Proportion meta-analysis estimating the rates of seroconversion following the second dose of TAK-003 vaccine in seronegative children/adolescents – DENV-4 serotype.

Study	ES	[95% Conf. Interval]	
Lopez-Medina (2022)	1.000	0.995	1.000
Biswal (2021)	0.996	0.977	0.999
Random pooled ES	1.000	0.997	1.000

Test of ES=0 : z= 92.923 p = 0.000

Table S44. Proportion meta-analysis estimating the rates of seroconversion following the second dose of TAK-003 vaccine in seronegative adults - All serotypes.

Study	ES	[95% Conf. Interval]	
Tricou V A 2023 (202	0.909	0.804	0.961
Tricou PLOS 2023 (20	0.980	0.964	0.989
Rupp (2015)	0.360	0.202	0.555
Tricou V B 2023 (202	0.956	0.930	0.973
Random pooled ES	0.877	0.742	0.969

Heterogeneity chi^2 = 63.018 (d.f. = 3) p = 0.000
I^2 (variation in ES attributable to heterogeneity) = 95.239%
Estimate of between-study variance Tau^2 = 0.108

Test of ES=0 : z= 13.091 p = 0.000

Table S45. Proportion meta-analysis estimating the rates of seroconversion following the second dose of TAK-003 vaccine in seronegative adults – DENV-1 serotype.

Study	ES	[95% Conf. Interval]	
Osorio (2014)	0.947	0.754	0.991
Tricou V A 2023 (202	1.000	0.935	1.000
Tricou PLOS 2023 (20	0.996	0.987	0.999
Takeda NCT03423173 (	0.978	0.958	0.989
Random pooled ES	0.994	0.975	1.000

Heterogeneity chi^2 = 8.945 (d.f. = 3) p = 0.030
I^2 (variation in ES attributable to heterogeneity) = 66.461%
Estimate of between-study variance Tau^2 = 0.011

Test of ES=0 : z= 39.558 p = 0.000

Table S46. Proportion meta-analysis estimating the rates of seroconversion following the second dose of TAK-003 vaccine in seronegative adults – DENV-2 serotype.

Study	ES	[95% Conf. Interval]	
Osorio (2014)	0.789	0.567	0.915
Tricou V A 2023 (202	1.000	0.935	1.000
Tricou PLOS 2023 (20	0.993	0.981	0.997
Takeda NCT03423173 (	0.992	0.976	0.997
Random pooled ES	0.991	0.964	1.000

Heterogeneity chi^2 = 12.526 (d.f. = 3) p = 0.006
I^2 (variation in ES attributable to heterogeneity) = 76.050%
Estimate of between-study variance Tau^2 = 0.017

Test of ES=0 : z= 33.011 p = 0.000

Table S47. Proportion meta-analysis estimating the rates of seroconversion following the second dose of TAK-003 vaccine in seronegative adults – DENV-3 serotype.

```
           Study     |     ES    [95% Conf. Interval]
---------------------+---------------------------------------------------
Osorio (2014)        |     0.947       0.754        0.991
Tricou V A 2023 (202 |     0.927       0.827        0.971
Tricou PLOS 2023 (20 |     0.989       0.976        0.995
Takeda NCT03423173 ( |     0.967       0.944        0.981
---------------------+---------------------------------------------------
Random pooled  ES    |     0.975       0.942        0.996
---------------------+---------------------------------------------------

  Heterogeneity chi^2 =     10.840 (d.f. = 3) p =      0.013
  I^2 (variation in ES attributable to heterogeneity) =    72.324%
  Estimate of between-study variance Tau^2 =      0.014

  Test of ES=0 : z=     34.045 p =      0.000
```

Table S48. Proportion meta-analysis estimating the rates of seroconversion following the second dose of TAK-003 vaccine in seronegative adults – DENV-4 serotype.

```
           Study     |     ES    [95% Conf. Interval]
---------------------+---------------------------------------------------
Osorio (2014)        |     0.737       0.512        0.882
Tricou V A 2023 (202 |     0.964       0.877        0.990
Tricou PLOS 2023 (20 |     0.987       0.974        0.994
Takeda NCT03423173 ( |     0.984       0.965        0.992
---------------------+---------------------------------------------------
Random pooled  ES    |     0.972       0.930        0.997
---------------------+---------------------------------------------------

  Heterogeneity chi^2 =     15.522 (d.f. = 3) p =      0.001
  I^2 (variation in ES attributable to heterogeneity) =    80.673%
  Estimate of between-study variance Tau^2 =      0.023

  Test of ES=0 : z=     28.399 p =      0.000
```

Table S49. Proportion meta-analysis estimating the rates of seroconversion following the second dose of TAK-003 vaccine in seropositive or seronegative (mixed) subjects of all ages (adults and children/adolescents) - All serotypes.

```
           Study     |     ES    [95% Conf. Interval]
---------------------+---------------------------------------------------
Tricou L 2020 (2020) |     0.916       0.836        0.959
Takeda NCT02948829 ( |     0.971       0.934        0.988
Sirivichayakul AD 20 |     0.848       0.718        0.924
Sirivichayakul CH 20 |     0.951       0.912        0.973
---------------------+---------------------------------------------------
Random pooled  ES    |     0.937       0.889        0.973
---------------------+---------------------------------------------------

  Heterogeneity chi^2 =      9.481 (d.f. = 3) p =      0.024
  I^2 (variation in ES attributable to heterogeneity) =    68.359%
  Estimate of between-study variance Tau^2 =      0.019

  Test of ES=0 : z=     29.804 p =      0.000
```

Table S50. Proportion meta-analysis estimating the rates of seroconversion following the second dose of TAK-003 vaccine in seropositive or seronegative (mixed) subjects of all ages (adults and children/adolescents) – DENV-1 serotype.

Study	ES	[95% Conf. Interval]	
Lopez-Medina (2022)	1.000	0.998	1.000
Takeda NCT02948829 (	0.995	0.970	0.999
Turner (2020)	0.982	0.966	0.991
Sirivichayakul AD 20	1.000	0.923	1.000
Sirivichayakul CH 20	1.000	0.981	1.000
George (2015)	0.778	0.453	0.937
Patel (2023)	0.993	0.960	0.999
Takeda NCT04313244 (	1.000	0.984	1.000
Random pooled ES	1.000	0.991	1.000

Heterogeneity chi^2 = 39.129 (d.f. = 7) p = 0.000
I^2 (variation in ES attributable to heterogeneity) = 82.110%
Estimate of between-study variance Tau^2 = 0.016

Test of ES=0 : z= 52.514 p = 0.000

Table S51. Proportion meta-analysis estimating the rates of seroconversion following the second dose of TAK-003 vaccine in seropositive or seronegative (mixed) subjects of all ages (adults and children/adolescents) – DENV-2 serotype.

Study	ES	[95% Conf. Interval]	
Lopez-Medina (2022)	1.000	0.998	1.000
Takeda NCT02948829 (	1.000	0.979	1.000
Turner (2020)	1.000	0.992	1.000
Sirivichayakul AD 20	0.978	0.887	0.996
Sirivichayakul CH 20	1.000	0.981	1.000
George (2015)	1.000	0.701	1.000
Patel (2023)	1.000	0.973	1.000
Takeda NCT04313244 (	0.996	0.977	0.999
Random pooled ES	1.000	1.000	1.000

Heterogeneity chi^2 = 10.212 (d.f. = 7) p = 0.177
I^2 (variation in ES attributable to heterogeneity) = 31.451%
Estimate of between-study variance Tau^2 = 0.002

Test of ES=0 : z= 109.859 p = 0.000

Table S52. Proportion meta-analysis estimating the rates of seroconversion following the second dose of TAK-003 vaccine in seropositive or seronegative (mixed) subjects of all ages (adults and children/adolescents) – DENV-3 serotype.

Study	ES	[95% Conf. Interval]	
Lopez-Medina (2022)	1.000	0.998	1.000
Takeda NCT02948829 (	1.000	0.979	1.000
Turner (2020)	0.989	0.974	0.995
Sirivichayakul AD 20	1.000	0.923	1.000
Sirivichayakul CH 20	0.995	0.973	0.999
George (2015)	1.000	0.701	1.000
Patel (2023)	0.978	0.938	0.993
Takeda NCT04313244 (	1.000	0.984	1.000
Random pooled ES	1.000	0.995	1.000

```
Heterogeneity chi^2 =     27.937 (d.f. = 7) p =      0.000
I^2 (variation in ES attributable to heterogeneity) =    74.943%
Estimate of between-study variance Tau^2 =      0.010
Test of ES=0 : z=     61.924 p =      0.000
```

Table S53. Proportion meta-analysis estimating the rates of seroconversion following the second dose of TAK-003 vaccine in seropositive or seronegative (mixed) subjects of all ages (adults and children/adolescents) – DENV-4 serotype.

Study	ES	[95% Conf. Interval]	
Lopez-Medina (2022)	1.000	0.998	1.000
Takeda NCT02948829 (	1.000	0.979	1.000
Turner (2020)	0.793	0.753	0.828
Sirivichayakul AD 20	0.826	0.693	0.909
Sirivichayakul CH 20	0.946	0.906	0.969
George (2015)	0.889	0.565	0.980
Patel (2023)	0.993	0.960	0.999
Takeda NCT04313244 (	1.000	0.984	1.000
Random pooled ES	0.967	0.883	1.000

```
Heterogeneity chi^2 =    381.170 (d.f. = 7) p =      0.000
I^2 (variation in ES attributable to heterogeneity) =    98.164%
Estimate of between-study variance Tau^2 =      0.185
Test of ES=0 : z=     16.409 p =      0.000
```

Table S54. Proportion meta-analysis estimating the rates of seroconversion following the second dose of TAK-003 vaccine in seropositive or seronegative (mixed) children/adolescents - All serotypes.

Study	ES	[95% Conf. Interval]	
Tricou L 2020 (2020)	0.916	0.836	0.959
Takeda NCT02948829 (	0.971	0.934	0.988
Sirivichayakul CH 20	0.951	0.912	0.973
Random pooled ES	0.952	0.921	0.976

```
Heterogeneity chi^2 =      3.551 (d.f. = 2) p =      0.169
I^2 (variation in ES attributable to heterogeneity) =    43.673%
Estimate of between-study variance Tau^2 =      0.005
Test of ES=0 : z=     40.697 p =      0.000
```

Table S55. Proportion meta-analysis estimating the rates of seroconversion following the second dose of TAK-003 vaccine in seropositive or seronegative (mixed) children/adolescents – DENV-1 serotype.

```
          Study     |     ES    [95% Conf. Interval]
---------------------+---------------------------------------------------
Lopez-Medina (2022)  |     1.000       0.998        1.000
Takeda NCT02948829 ( |     0.995       0.970        0.999
Sirivichayakul CH 20 |     1.000       0.981        1.000
Takeda NCT04313244 ( |     1.000       0.984        1.000
---------------------+---------------------------------------------------
Random pooled  ES    |     1.000       0.998        1.000
---------------------+---------------------------------------------------

  Heterogeneity chi^2 =      4.801 (d.f. = 3) p =      0.187
  I^2 (variation in ES attributable to heterogeneity) =    37.515%
  Estimate of between-study variance Tau^2 =      0.002

  Test of ES=0 : z=     93.024 p =      0.000
```

Table S56. Proportion meta-analysis estimating the rates of seroconversion following the second dose of TAK-003 vaccine in seropositive or seronegative (mixed) children/adolescents – DENV-2 serotype.

```
          Study     |     ES    [95% Conf. Interval]
---------------------+---------------------------------------------------
Lopez-Medina (2022)  |     1.000       0.998        1.000
Takeda NCT02948829 ( |     1.000       0.979        1.000
Sirivichayakul CH 20 |     1.000       0.981        1.000
Takeda NCT04313244 ( |     0.996       0.977        0.999
---------------------+---------------------------------------------------
Random pooled  ES    |     1.000       0.998        1.000
---------------------+---------------------------------------------------

  Heterogeneity chi^2 =      4.603 (d.f. = 3) p =      0.203
  I^2 (variation in ES attributable to heterogeneity) =    34.820%
  Estimate of between-study variance Tau^2 =      0.001

  Test of ES=0 : z=     95.977 p =      0.000
```

Table S57. Proportion meta-analysis estimating the rates of seroconversion following the second dose of TAK-003 vaccine in seropositive or seronegative (mixed) children/adolescents – DENV-3 serotype.

```
          Study     |     ES    [95% Conf. Interval]
---------------------+---------------------------------------------------
Lopez-Medina (2022)  |     1.000       0.998        1.000
Takeda NCT02948829 ( |     1.000       0.979        1.000
Sirivichayakul CH 20 |     0.995       0.973        0.999
Takeda NCT04313244 ( |     1.000       0.984        1.000
---------------------+---------------------------------------------------
Random pooled  ES    |     1.000       0.998        1.000
---------------------+---------------------------------------------------

  Heterogeneity chi^2 =      4.724 (d.f. = 3) p =      0.193
  I^2 (variation in ES attributable to heterogeneity) =    36.496%
  Estimate of between-study variance Tau^2 =      0.002

  Test of ES=0 : z=     94.125 p =      0.000
```

Table S58. Proportion meta-analysis estimating the rates of seroconversion following the second dose of TAK-003 vaccine in seropositive or seronegative (mixed) children/adolescents – DENV-4 serotype.

Study	ES	[95% Conf.	Interval]
Lopez-Medina (2022)	1.000	0.998	1.000
Takeda NCT02948829 (	1.000	0.979	1.000
Sirivichayakul CH 20	0.946	0.906	0.969
Takeda NCT04313244 (	1.000	0.984	1.000
Random pooled ES	0.996	0.972	1.000

```
Heterogeneity chi^2 =     39.823 (d.f. = 3) p =      0.000
I^2 (variation in ES attributable to heterogeneity) =    92.467%
Estimate of between-study variance Tau^2 =      0.034

Test of ES=0 : z=     30.160 p =      0.000
```

Table S59. Proportion meta-analysis estimating the rates of seroconversion following the second dose of TAK-003 vaccine in seropositive or seronegative (mixed) adults – DENV-1 serotype.

Study	ES	[95% Conf.	Interval]
Turner (2020)	0.982	0.966	0.991
Sirivichayakul AD 20	1.000	0.923	1.000
George (2015)	0.778	0.453	0.937
Patel (2023)	0.993	0.960	0.999
Random pooled ES	0.995	0.969	1.000

```
Heterogeneity chi^2 =      6.949 (d.f. = 3) p =      0.074
I^2 (variation in ES attributable to heterogeneity) =    56.828%
Estimate of between-study variance Tau^2 =      0.013

Test of ES=0 : z=     33.013 p =      0.000
```

Table S60. Proportion meta-analysis estimating the rates of seroconversion following the second dose of TAK-003 vaccine in seropositive or seronegative (mixed) adults – DENV-2 serotype.

Study	ES	[95% Conf.	Interval]
Turner (2020)	1.000	0.992	1.000
Sirivichayakul AD 20	0.978	0.887	0.996
George (2015)	1.000	0.701	1.000
Patel (2023)	1.000	0.973	1.000
Random pooled ES	1.000	0.998	1.000

```
Heterogeneity chi^2 =      4.551 (d.f. = 3) p =      0.208
I^2 (variation in ES attributable to heterogeneity) =    34.077%
Estimate of between-study variance Tau^2 =      0.005

Test of ES=0 : z=     46.090 p =      0.000
```

Table S61. Proportion meta-analysis estimating the rates of seroconversion following the second dose of TAK-003 vaccine in seropositive or seronegative (mixed) adults – DENV-3 serotype.

Study	ES	[95% Conf. Interval]	
Turner (2020)	0.989	0.974	0.995
Sirivichayakul AD 20	1.000	0.923	1.000
George (2015)	1.000	0.701	1.000
Patel (2023)	0.978	0.938	0.993
Random pooled ES	0.998	0.989	1.000

Heterogeneity chi^2 = 1.467 (d.f. = 3) p = 0.690
I^2 (variation in ES attributable to heterogeneity) = 0.000%
Estimate of between-study variance Tau^2 = 0.000

Test of ES=0 : z= 69.299 p = 0.000

Table S62. Proportion meta-analysis estimating the rates of seroconversion following the second dose of TAK-003 vaccine in seropositive or seronegative (mixed) adults – DENV-4 serotype.

Study	ES	[95% Conf. Interval]	
Turner (2020)	0.793	0.753	0.828
Sirivichayakul AD 20	0.826	0.693	0.909
George (2015)	0.889	0.565	0.980
Patel (2023)	0.993	0.960	0.999
Random pooled ES	0.897	0.712	0.998

Heterogeneity chi^2 = 59.246 (d.f. = 3) p = 0.000
I^2 (variation in ES attributable to heterogeneity) = 94.936%
Estimate of between-study variance Tau^2 = 0.188

Test of ES=0 : z= 9.735 p = 0.000

Study or Subgroup	Vaccine Events	Vaccine Total	Placebo Events	Placebo Total	Weight	Risk Ratio M-H, Random, 95% CI	Year
**1.1.1 Children and adolescents**							
Tricou Lancet 2020	7	200	13	198	1.8%	0.53 [0.22, 1.31]	2020
Sirivichayakul (CH) 2022	3	203	2	83	0.5%	0.61 [0.10, 3.60]	2022
Tricou Lancet 2024	442	13380	547	6687	97.6%	0.40 [0.36, 0.46]	2024
**Subtotal (95% CI)**		**13783**		**6968**	**99.9%**	**0.41 [0.36, 0.46]**	
Total events	452		562				
Heterogeneity: Tau² = 0.00; Chi² = 0.57, df = 2 (P = 0.75); I² = 0%							
Test for overall effect: Z = 14.64 (P < 0.00001)							
**1.1.2 Adults**							
Sirivichayakul (AD) 2022	1	46	0	28	0.1%	1.85 [0.08, 43.94]	2022
**Subtotal (95% CI)**		**46**		**28**	**0.1%**	**1.85 [0.08, 43.94]**	
Total events	1		0				
Heterogeneity: Not applicable							
Test for overall effect: Z = 0.38 (P = 0.70)							
**Total (95% CI)**		**13829**		**6996**	**100.0%**	**0.41 [0.36, 0.46]**	
Total events	453		562				
Heterogeneity: Tau² = 0.00; Chi² = 1.45, df = 3 (P = 0.69); I² = 0%							
Test for overall effect: Z = 14.61 (P < 0.00001)							
Test for subgroup differences: Chi² = 0.88, df = 1 (P = 0.35), I² = 0%							

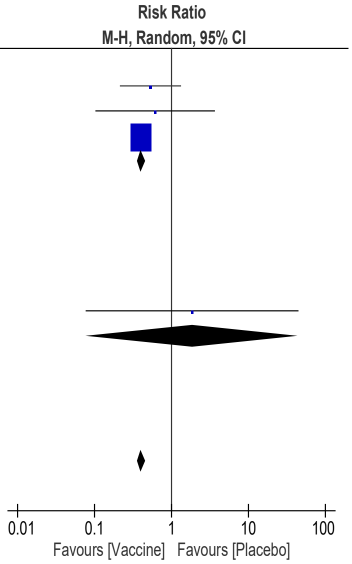

Figure S1. Risk of virologically-confirmed Dengue fever among vaccinated vs. control subjects in the overall sample and by age class.

A. DENV-1 serotype.

Study or Subgroup	Vaccine Events	Vaccine Total	Placebo Events	Placebo Total	Risk Ratio M-H, Random, 95% CI	Year
Tricou Lancet 2020	5	200	5	198	0.99 [0.29, 3.37]	2020
Tricou Lancet 2024	222	13377	230	6686	0.48 [0.40, 0.58]	2024
**Total (95% CI)**		**13577**		**6884**	**0.53 [0.33, 0.85]**	
Total events	227		235			

Heterogeneity: Tau² = 0.06; Chi² = 1.30, df = 1 (P = 0.25); I² = 23%
Test for overall effect: Z = 2.62 (P = 0.009)

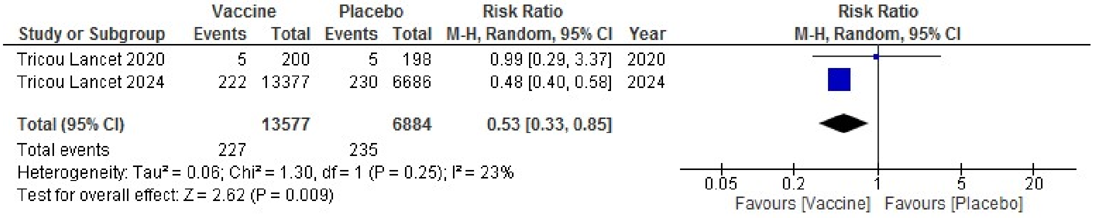

B. DENV-2 serotype.

Study or Subgroup	Vaccine Events	Vaccine Total	Placebo Events	Placebo Total	Risk Ratio M-H, Random, 95% CI	Year
Tricou Lancet 2020	0	200	5	198	0.09 [0.01, 1.62]	2020
Tricou Lancet 2024	68	13377	193	6686	0.18 [0.13, 0.23]	2024
**Total (95% CI)**		**13577**		**6884**	**0.18 [0.13, 0.23]**	
Total events	68		198			

Heterogeneity: Tau² = 0.00; Chi² = 0.21, df = 1 (P = 0.65); I² = 0%
Test for overall effect: Z = 12.48 (P < 0.00001)

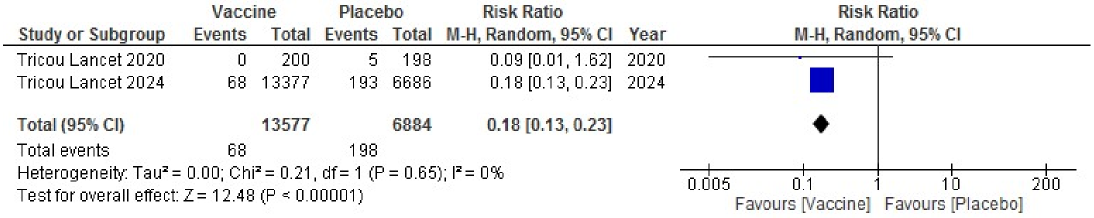

C. DENV-3 serotype.

Study or Subgroup	Vaccine Events	Vaccine Total	Placebo Events	Placebo Total	Risk Ratio M-H, Random, 95% CI	Year
Tricou Lancet 2020	1	200	3	198	0.33 [0.03, 3.15]	2020
Tricou Lancet 2024	132	13377	113	6686	0.58 [0.45, 0.75]	2024
**Total (95% CI)**		**13577**		**6884**	**0.58 [0.45, 0.74]**	
Total events	133		116			

Heterogeneity: Tau² = 0.00; Chi² = 0.24, df = 1 (P = 0.62); I² = 0%
Test for overall effect: Z = 4.31 (P < 0.0001)

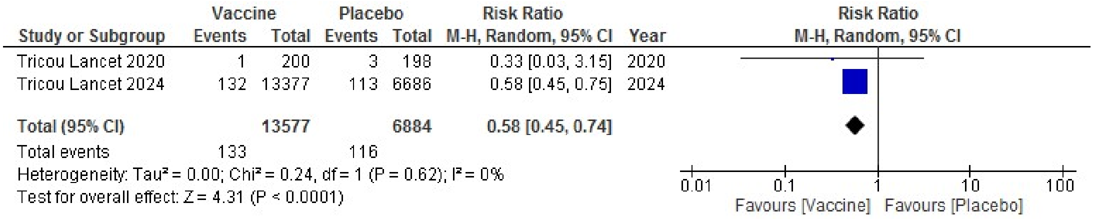

D. DENV-4 serotype.

Study or Subgroup	Vaccine Events	Vaccine Total	Placebo Events	Placebo Total	Risk Ratio M-H, Random, 95% CI	Year
Tricou Lancet 2020	1	200	1	198	0.99 [0.06, 15.72]	2020
Tricou Lancet 2024	24	13377	23	6686	0.52 [0.29, 0.92]	2024
**Total (95% CI)**		**13577**		**6884**	**0.54 [0.31, 0.94]**	
Total events	25		24			

Heterogeneity: Tau² = 0.00; Chi² = 0.20, df = 1 (P = 0.66); I² = 0%
Test for overall effect: Z = 2.19 (P = 0.03)

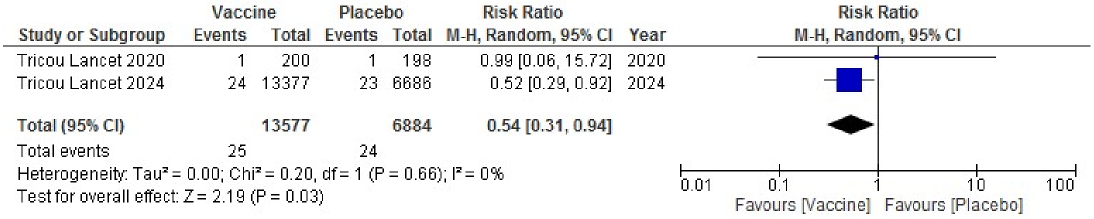

Figure S2. Risk of virologically confirmed Dengue fever among vaccinated vs. control subjects stratified by DENV serotype.

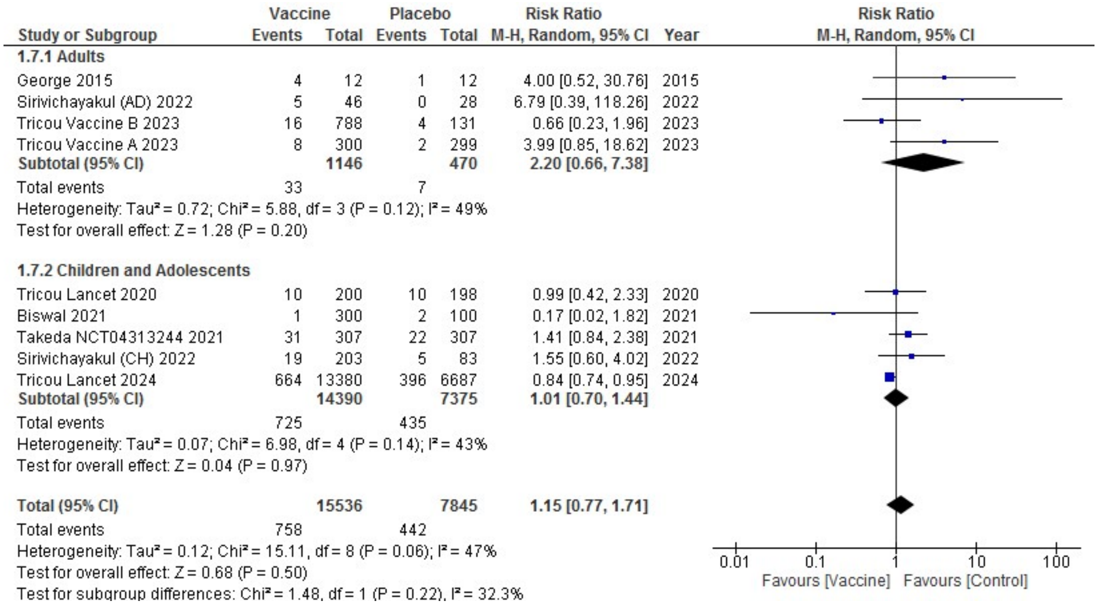

Figure S3. Risk of any serious adverse events (SAEs) among vaccinated vs. control subjects in the overall sample and by age class.

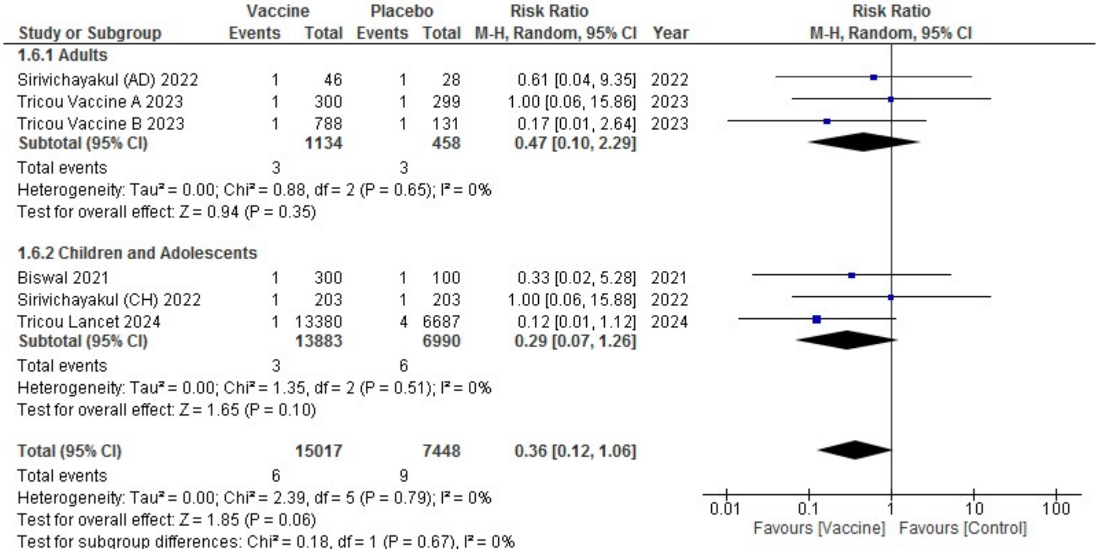

Figure S4. Risk of product-related serious adverse events (SAEs) among vaccinated vs. control subjects in the overall sample and by age class. Only the study by Tricou (Lancet 2024) reported one and four product-related SAEs, all other studies reported zero events. One event was thus added in each study cell to enable the meta-analysis.
